# Estimating ideology and polarization in European countries using Facebook data

**DOI:** 10.1140/epjds/s13688-022-00367-1

**Published:** 2022-11-22

**Authors:** Francisco Caravaca, José González-Cabañas, Ángel Cuevas, Rubén Cuevas

**Affiliations:** 1 Department of Telematic Engineering, Universidad Carlos III de Madrid, Avenida de la Universidad 30 Building 4, 28911 Leganés, Spain; 2 UC3M-Santander Big Data Institute, Calle Madrid 135 Building 18, 28903 Getafe, Spain

**Keywords:** Europe, Politics, Barometer, Ideology, Polarization, Facebook

## Abstract

Researchers have studied political ideology and polarization in many different contexts since their effects are usually closely related to aspects and actions of individuals and societies. Hence, being able to estimate and measure the changes in political ideology and polarization is crucial for researchers, stakeholders, and the general public. In this paper, we model the ideology and polarization of 28 countries (the 27 EU member states plus the UK) using Facebook public posts from political parties’ Facebook pages. We collected a three-year dataset from 2019 to 2021 with information from 234 political parties’ Facebook pages and took advantage of the EU parliament elections of May 2019 to create our models. Our methodology works across 28 countries and benefits from being a low-cost running process that measures ideology and polarization at a high-resolution time scale. The results show our models are pretty accurate when validating them against 19 individual countries’ elections as ground truth. Moreover, to make our results available to the research community, stakeholders, and individuals interested in politics, the last contribution of our paper is a website including detailed information about the political parties in our dataset. It also includes the temporal evolution of our ideology and polarization estimations. Therefore, our work delivers a novel tool that uses Facebook public data to create country metrics useful for different purposes. To the best of our knowledge, there is no prior work in the literature offering a solution that measures the ideology and polarization of all EU + UK countries.

## Introduction

The political ideology and polarization of a country are tightly linked to aspects related to the development of a society. This has attracted the interest of the research community to study the effect of ideology and polarization in society. We can find works exploring the impact of political ideology on the economic growth of a country [1–3] or discussing whether the level of education may impact the ideology of an individual [4–7]. Some works provide evidence on how the ideology of individuals also affects the trust in science [8, 9], the attitudes toward environmental decisions [10], climate change [11], fashion trends [12], consumption preferences [13], etc. Similarly, the literature has proved that political polarization is closely related to other socioeconomic variables such as the economic expectations [14] or the corruption perception in a country [15]. Some works also claim that polarization may improve the level of democracy [16]. For instance, the degree of polarization of a country could affect the voters’ turnout [17, 18]. Finally, recent works have also analyzed the impact of political ideology and polarization on the COVID-19 pandemic [19–22].

This large body of research shows that measuring political ideology and polarization is of great importance (directly) for the research community to tackle multiple socio-economic issues and (indirectly) for policymakers that can use the output of the literature to take action to improve the quality of societies. For this reason, it is crucial to have efficient methodologies to measure political ideology and polarization.

The most widespread way to measure ideology and polarization relies on defining ad-hoc surveys, interviews, polls, etc., and processing the collected information to derive a quantitative metric for ideology or polarization in a given country or region. Public opinion research entities run surveys regularly for a specific country or region, often electoral polls (e.g., once a month or quarter), in most of the countries or a broader scope including multiple countries or regions. Examples of the latter are the World Value Survey (WVS) [23], the European Value Survey (EVS) [24] or the European Quality of Government Index survey (EQGI) [25]. Although the quality of these surveys is not under discussion since they are of great value for the research community, policymakers, and other stakeholders, they have some limitations. First of all, all these surveys, independently whether they are run for a single or multiple country/regions are expensive. Second, they usually present either low geographical and/or temporal resolutions. On the one hand, surveys run frequently (e.g., electoral polls) and usually focus on a single country or region. Expanding them further will very likely imply substantially higher costs and time. On the other hand, surveys with a large geographical coverage usually have a limited temporal resolution. For instance, the WVS, EVS, or EQGI are released every 4 years.

We believe that there is room to explore novel alternatives to surveys that allow us to achieve the three requirements implicitly discussed before: large geographical coverage, low temporal resolution, and low cost. This paper aims to propose a novel methodology using information publicly available on the Facebook (FB) pages of political parties (e.g., number of followers, posts published, posts reactions, etc.) to predict political ideology and polarization across the 27 countries forming the European Union (EU) plus the United Kingdom (UK), i.e., the 28 EU members before Brexit. We clarify that in the case of polarization we are actually focusing on party system polarization as a way to measure the distribution of parties in the left-right dimension in a parliament. The underlying idea is to understand whether we can effectively predict ideology and polarization with data that roughly captures: political parties’ popularity (number of followers), political party activity (posting frequency), and users’ engagement (reactions such as likes, shares, or comments). Note, that the reason to select users’ engagement [26–30], popularity [27, 31] and activity [28, 32, 33] is because previous work in the literature have studied the relationship between those metrics from social media and elements around electoral results. In case we succeed in generating an accurate prediction model we will meet the three pre-defined requirements. First, our methodology is designed to cover a large geographical area including 28 different countries. Second, our methodology is designed to provide results at a very high frequency (e.g., every week) since the data for our model is readily available. Third, our methodology is inexpensive since it relies on publicly available data that can be collected and processed at a low cost. In addition, our solution allows using a single data source and methodology to compute the polarization and ideology in parallel in all EU countries, which is of great interest to perform comparative studies. Furthermore, the high temporal resolution offered by this methodology enables the possibility of studying the ideology and polarization evolution over time with fine granularity. Finally, the methodology is extensible beyond the EU + UK. In a nutshell, the main contribution of this paper is defining a methodology that allows computing the political ideology and polarization in each of the 28 EU + UK countries at a low cost and high frequency. To the best of our knowledge, there is no prior work that meets these requirements in a large geographical scope such as the EU + UK.

The core of the proposed methodology is the definition of a model to predict the political ideology and the party polarization of a country. To create that model, we have first obtained the data from 234 FB pages belonging to the most important political parties in each of the 28 countries considered in our study between Jan 1. 2019, and Dec. 31. 2021. For each of these pages, we collect all the posts publicly available along with their timestamp, the number of reactions attracted by each post (likes, shares, and comments), and the total number of followers of the page. Second, we study what are the best variables available in our dataset to build the model Third, we compute ideology and polarization metrics with the variables collected using them as a proxy of party vote share for each country, with the possibility of computing them in different time windows. Fourth, we apply a regression model based on the previous metrics between Jan 2019 and May 2019 because we used the May 2019 EU parliament election, where all the 28 countries were involved, as ground truth for training our model. Finally, to evaluate and validate the performance and accuracy of our model, we use national elections of 15 EU countries that took place between 2019 and 2020 and 4 elections run in 2016. The results show that the proposed models of ideology and polarization display good accuracy. This means that they can be used as a good proxy to measure these political parameters meeting the three requirements we imposed. Our findings suggest that the post activity and the comments received in posts are good indicators to predict ideology, whereas the post activity and reactions received in posts are the best features to estimate polarization. The proposed methodology allows monitoring the evolution of the ideology and polarization over time in small intervals.

We also evaluated the performance of the ideology and polarization models to predict parties’ vote share. We obtain an average Mean Average Error (MAE) higher than 8 (i.e., 8 percentage points). As we expected, a model created from a few metrics extracted from FB posts posted on political parties’ pages is not accurate enough to predict parties’ vote share.

The second contribution of this paper is the implementation of a barometer that leverages the output of our model to monitor the evolution of the political ideology and polarization of the 28 countries in our dataset. The barometer has been implemented as an interactive website that displays the ideology and polarization of the political parties and countries over time. The website allows selecting only one party/country or comparing multiple parties/countries in the same visualization. The site also provides aggregated information on the activity and engagement in FB across all parties used in this study. Finally, we adopt an open science approach making the data used to generate the polarization and ideology graphs available to be downloaded and used by other researchers and stakeholders.

To conclude the introduction section, we would like to state that the goal of this paper is not replacing traditional methods to measure political ideology, but propose a novel approach that can coexist with traditional ones and provide other beneficial features (i.e., low-cost, high temporal and spatial resolution, etc.) that may be of interest for studies that require fine-grained temporal data across many countries.

## Dataset

In this section, we introduce the data collection process and the resulting dataset. We use this information for a twofold purpose. First, to create a regression model to quantify the political ideology and polarization for each of the EU + UK countries based on the information related to the Facebook posts from political parties in these countries. Second, to create an interactive website that implements a Eurobarometer that monitors the political ideology and polarization within the EU + UK countries. After introducing the data collection and dataset, we also present a few interesting insights out of a preliminary analysis of the data.

### Dataset collection and description

We use two different data collection processes to create a complete dataset used for implementing the model that predicts political ideology and polarization. On the one hand, we have used a publicly available repository to retrieve the political ideology assigned to the political parties within the 28 countries covered in this paper. On the other hand, we have collected the information from the FB pages associated with those political parties including the number of followers, the posts published, and the users’ reactions to these posts. Following, we describe in detail each of the two processes.

#### Political parties and ideology dataset

We relied on an external geopolitical open database to retrieve a comprehensive list of EU + UK political parties for our study along with their political ideology. We did an intensive search and evaluated multiple open databases such as the Global Party Survey 2019 [34], V-Party [35], or ParlGov [36], which provide political information for many countries. We selected ParlGov because it includes the best information for the goal of our research. Overall, the ParlGov database includes 1294 political party entries within the EU and UK.^1^ The main reasons to select ParlGov are: $(i)$ it includes an extensive list of political parties within the EU + UK; $(\mathit{ii})$ it provides the ideology for each political party using the standard left-right scale [37]. This scale ranges between 0 (extreme left) and 10 (extreme right). It is important to note that we assumed the ideology of a party is time-invariant in the period considered in our work; $(\mathit{iii})$ ParlGov provides relevant information about the electoral events in the 28 EU + UK countries. We use the electoral results to train and evaluate the performance of our prediction models.

#### Facebook parties’ page selection

To create the dataset, the first step was mapping the EU + UK political parties included in ParlGov to their official main Facebook accounts. This was not an easy step since it required carrying out manual verification in many of the analyzed countries. For instance, some parties have multiple official pages that include the main page of the party but also Facebook pages belonging to regional sections of the party, or particular groups within the party such as young associations. In this paper, we only take into consideration the information from the main page of each party because $(i)$ in the vast majority, the main page is the one attracting the most attention in terms of followers, page likes, and reactions to posts; $(\mathit{ii})$ the main page of the party is the one that best captures the political messages the organization wants to disseminate to the overall society, $(\mathit{iii})$ even though in some cases the FB page of the political leader of a party attracts more attention than the main page of the party, we do not consider them for our study, as the leaders of parties could change at any point. For instance, since the last Spanish elections in Nov. 2019 3 out of the 5 leaders of major parties have changed. Altogether, out of the 1294 political parties retrieved from ParlGov in EU + UK, we were able to find the official Facebook main page for 246 of them, out of which 234 have an assigned value on the left-right scale.

Our goal is to represent the highest amount of vote share possible in each country, some small parties may not be represented, but we had made an exhaustive search for every party that represents at least 5% of the vote share in recent elections. Out of the 159 parties with a left-right value and more than 5% of votes in the 2019 EU elections, we collected information from 153 parties. Altogether, the 246 parties received (in average) the 90.45% of the votes in the 2019 EU Elections (see the disaggregated values per country in Table 1). All the countries’ parties we considered represent above 65% of votes, and all but Croatia and Greece above 75%. Overall, our FB dataset is very comprehensive and includes the vast majority of relevant EU parties. The remaining parties within the ParlGov dataset are either very minor parties, inactive, or simply do not exist anymore.

**Table 1:** Number of posts and political parties in our dataset from each country

Country	Parties	EU Elec. (%)	Posts	Country-PPD	Country-SD	Party-PPD	Party-SD
Austria	5	98.20	14,544	13.270	6.498	2.654	0.708
Belgium	12	97.85	21,494	19.611	8.391	1.634	0.528
Bulgaria	10	91.14	24,261	22.156	12.533	3.315	1.116
Croatia	11	69.08	18,781	17.183	11.494	2.515	0.748
Cyprus	7	84.18	21,329	19.461	13.863	2.780	0.988
Czech Republic	7	88.59	19,674	17.951	9.335	2.564	1.044
Denmark	10	100.00	11,976	10.927	6.933	1.093	0.386
Estonia	6	88.70	12,976	11.839	6.157	1.973	0.254
Finland	9	96.80	18,113	16.526	10.732	1.836	0.667
France	10	90.01	21,345	19.475	12.598	1.948	0.828
Germany	8	85.42	16,883	15.404	7.085	2.290	0.709
Greece	7	73.32	19,498	17.790	10.212	2.541	1.809
Hungary	6	94.78	26,218	23.922	13.293	3.987	1.617
Ireland	8	76.07	11,536	10.526	7.166	1.316	0.667
Italy	7	94.67	42,399	38.685	36.807	5.526	7.304
Latvia	9	96.32	13,359	12.189	9.367	1.354	0.316
Lithuania	10	81.38	9518	9.359	6.162	1.992	0.435
Luxembourg	6	96.21	5144	4.693	3.707	0.782	0.203
Malta	4	94.22	6132	5.595	4.528	1.399	0.908
Netherlands	12	97.54	12,982	11.845	8.639	0.987	0.663
Poland	6	95.70	19,757	18.026	8.606	3.004	1.252
Portugal	8	91.58	27,058	24.688	15.181	3.086	2.591
Romania	8	93.43	22,681	20.694	15.799	2.587	1.273
Slovakia	11	82.37	23,315	21.273	10.024	1.934	0.827
Slovenia	9	93.22	17,415	15.890	9.062	1.766	0.851
Spain	11	91.18	36,006	32.852	29.488	2.987	1.447
Sweden	8	98.27	15,762	14.381	7.940	1.798	1.106
United Kingdom	9	92.50	20,174	18.407	10.900	2.045	0.715

EU Elec is the percentage of votes (2019 EU Elections) those parties (and the parties without a left-right value) obtained in the EU parliament 2019 elections per country. Country-PPD (Country Posts Per Day) represents the average amount of posts published in a country for each day, Country-SD measures its standard deviation. Party-PPD (Party Posts Per Day) is the mean of the PPD calculated for each party, and Party-SD its standard deviation.

**Figure 1:**
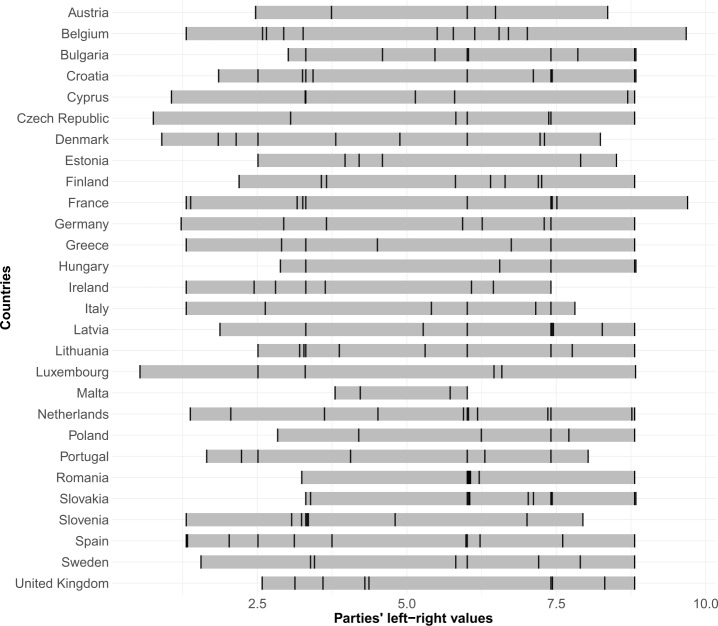
Political parties’ ideology distribution in European countries. Each vertical line represents the ideology of each political party

To illustrate the ParlGov left-right ideology distribution, Fig. 1 displays the left-right position of each of the 234 parties of our Facebook dataset. Each vertical line represents the ideology of each political party according to the value that ParlGov assigns them on the left-right scale. This figure depicts at a glance the political-ideological distribution of the main political Facebook parties across the EU and UK. We observe that the left-most party belongs to Luxembourg, *i.e.*, Déi Lénk, with an ideology value equal to 0.5263. On the opposite extreme, the two right-most parties are in France (*i.e.*, Rassemblement National) and Belgium (*i.e.*, Vlaams Blok), with values of 9.6854 and 9.6622, respectively. Finally, the number of political parties is quite heterogeneous across the countries in our dataset. In Table 1 we can see that Belgium and the Netherlands include the most political parties in our dataset (12), while Malta has the least (4).

#### Facebook dataset

**Figure 2:**
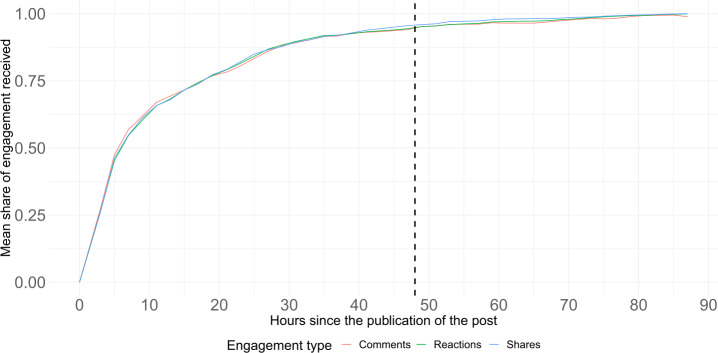
Engagement evolution after the publication of a post. The vertical line represents the 48 hours mark

We collect the information available within the main official Facebook pages of the political parties. These pages are public on Facebook and anyone can browse through their posts. For each party page on Facebook, we collect $(i)$ the number of followers on the page, $(\mathit{ii})$ the number of likes the page has received, $(\mathit{iii})$ the content (i.e., text, URL links, etc.) of the public posts published by the political parties in their FB page from January 1st, 2019 until December 31st, 2021, $(\mathit{iv})$ users’ engagement for each of the posts. This includes the number of reactions, shares, comments, and video views (in case the content of the post is a video) from the users. In particular, the reactions include multiple options such as *like, love, care, haha, wow, sad* and *angry*. We obtain the aggregated number of reactions as well as the specific number for each reaction type. We gather the value of each of the engagement variables for a post at least 48 hours after the post was published. We have observed that the engagement increase mainly occurs in the 48 hours after the post publication in the vast majority of posts, as shown in Fig. 2. Overall, our dataset is roughly capturing the popularity (i.e., followers and likes of the page), activity (number of posts published over time), and engagement (number of reactions, shares, and comments per post) of each political party under analysis. The relationship between these dimensions and the vote share received by a party in elections had been studied on Facebook, with different degrees of success [26, 27, 32], which could suggest that these indicators could be related to ideology and polarization, as the definitions of both ideology and polarization need the party vote share as an input, those definitions will be introduced in Sect. 3.1.

We created an automatic process to navigate through the parties’ pages and scrap the publicly available posts from the 234 FB pages in our list. Our process collected posts information for 3 complete years between January 2019 and December 2021. As a result of this, our dataset includes more than 500k posts across the 234 parties along with the left-right ideology ParlGov assigned to each of them.

### Insights of political parties activity in Facebook

Before moving to the main objective of this paper, i.e., creating the political ideology and polarization model, we briefly discuss a few interesting insights derived from a comparative analysis of the activity of the EU + UK political parties on Facebook. We look into four specific elements: (i) activity overtime referred to as *temporal analysis*; (ii) activity across countries referred to as *inter-country analysis*; (iii) activity across political parties within the same country referred to as *intra-country analysis*; and, (iv) activity per ideology *ideology analysis*. To carry out some of these analyses we rely on the results depicted in Table 1. This table shows the number of political parties considered in each country, the aggregated percentage of votes those parties obtained in the EU parliament 2019 elections per country, the overall number of posts collected per country, and the metrics associated with two different methods to measure the FB activity of political parties. The first one measures the aggregated activity of all parties by looking at the average number of posts per day published in each country (Country-PPD) along with its standard deviation (Country-SD). The second one measures the average posts per day published by a single political party in each country (Party-PPD) along with its standard deviation (Party-SD).

#### Temporal analysis of activity

**Figure 3:**
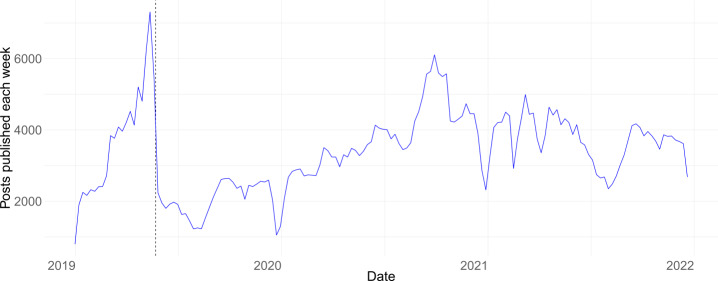
The total number of posts published. The vertical line represents the 2019 European Elections

Figure 3 shows the aggregated number of posts published per week across the 28 countries between Jan. 1, 2019, and Dec. 31, 2021. As we expected, the distribution of posts per week is not homogeneously distributed. Instead, we observe spikes and valleys.

We have included a vertical dashed line in the figure corresponding to the last EU parliament election days (23-26 May 2019) that ran in parallel in all the 28 countries analyzed in this paper because at that time UK was still an EU member. We observe that the largest peak happens in the week before the elections May 13-19, 2019. After the elections, the number of posts published reduced drastically.

The second-largest spike happens in autumn 2020, mainly due to the regional elections of Italy on September 20 and 21, with a relevant but smaller contribution from the Czech Republic regional elections on 2 and 3 October. Finally, we observe that Christmas week always shows a decrease in political party activity on Facebook in 2019, 2020, and 2021. We note that Appendix A.1 includes the figures for the number of posts per week for each of the 28 countries of our dataset. We observe the same pattern in the countries holding individual elections at different periods after the European elections.

The temporal activity results demonstrate that, as previously reported in the literature [38], political parties increase their activity before the electoral periods. This implicitly implies that political parties consider Facebook as a relevant channel to disseminate their messages.

#### Inter-country activity comparison

On average, we have collected 18,940 posts per country. Luxembourg is the country with the lowest number of posts (5144), and Italy is the one with the greatest number (42,399). Similarly, we observe a substantial difference in the intensity level of daily political posts across the 28 analyzed countries. Italy is the most active country with 38.7 posts per day on average, followed by Spain (32.9) and Portugal (24.7). Contrarily, the countries showing the lowest daily activity are Luxembourg (5.3), Malta (5.7), and Lithuania (9.4). The large standard deviation observed in the Country-PPD aligns with the results depicted in the temporal analysis since there are days/weeks with intense activity (mostly before electoral periods) and days of very low activities (e.g., Christmas).

To roughly understand whether the publishing intensity may respond to basic socio-economic parameters such as the population or wealth, we have computed the Pearson correlation between the Country-PPD and the population and GDP per capita of each country. The population shows a rather strong correlation of 0.447 with the Country-PPD, while the GDP per capita is in contrast negatively correlated with a person correlation equal to −0.395. Therefore, high population and low GDP per capita seem to be indicators of a higher activity of political parties in FB.

To understand why a lower GDP per capita may imply more Facebook activity, we have also computed the correlation between GDP per capita and country ideology obtaining a result of −0.37. This roughly means that countries with lower GDP per capita are more biased towards the right. As we will see in Sect. 2.2.4, left parties (a group of parties whose ideology is between 1.5 and 3.5) are less active on Facebook. Therefore, the observed correlation between GDP per capita and ideology may explain why countries with a lower GDP per capita are more active.

In a nutshell, our results show that the FB activity of political parties substantially diverges from country to country when considering the EU + UK.

#### Intra-country activity comparison

In this subsection, we analyze whether the posts are evenly distributed among the political parties when you consider a single country or instead a few parties are responsible for a large portion of all published posts. We leverage the Party-PPD and the Party-SD depicted in Table 1 to perform the analysis.

In most of the countries, the average publishing activity of parties (Party-PPD) is below 3, with 17 out of the 28 countries with a value between 1.5 and 3. Luxembourg is the country with the lowest Party-PPD with a value of 0.8 posts per day. Contrarily, Italian parties are the ones that publish the most on average, with a Party-PPD of 5.5.

The average Party-SD/Party-PPD ratio across the 28 countries is almost 0.5. The higher this ratio is, the more divergent the publication rate is across political parties in a country. Italy is the country showing the highest ratio (1.3) which means there may be some parties that publish many more FB posts than others. If we look in detail, we find that *Lega per Salvini Premier* is responsible for 55.95% of all the posts published in Italy in the analyzed period. Portugal and Greece are the countries showing the second and third largest divergence in the FB posting rate among their political parties (0.84 and 0.71, respectively). Contrarily, Estonia, Latvia, Luxembourg, and Austria show a rather homogeneous posting rate across parties with a Party-SD/Party-PPD ratio below 0.3.

In summary, the divergence among the FB publishing rate of political parties within a country largely varies from country to country in our dataset, with Italy representing the extreme case with a very dominant party.

#### Ideology-based activity comparison

The literature has evidenced the differences in the impact that ideological messages have on users depending on the spectrum of such messages in the *left-right* scale [39]. In this section, we aim to explore whether we can observe substantial differences in the aggregated activity of political parties according to the ideology assigned by ParlGov. This means, whether parties at a particular part of the political spectrum (e.g., left, central, right) post more messages or not.

**Figure 4:**
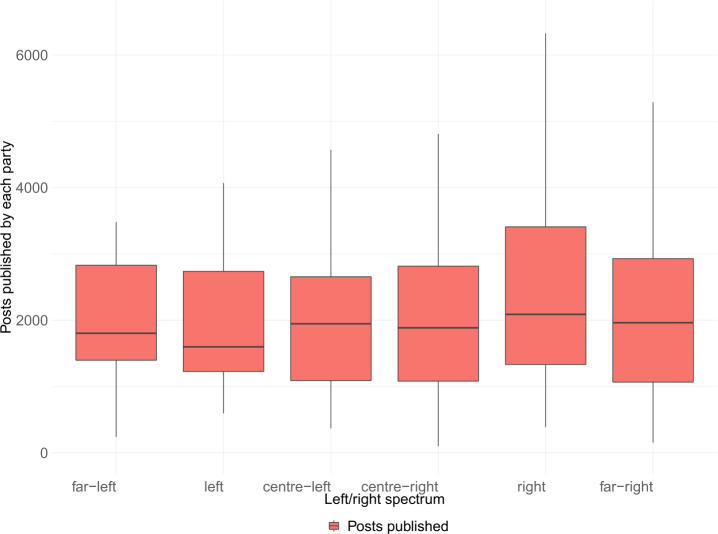
Boxplot of posts published by each party, divided by ideology. The ideology is divided in the left-right scale using the following intervals: far-left $0 < 1.5$, left $1.5 < 3.5$ centre-left $3.5 < 5$, centre-right $5 < 6.5$, right $6.5 < 8.5$, far-right $8.5 < 10$

Figure 4 displays a boxplot showing per each ideological position the distribution of the number of posts published per party for a particular ideology. We divided the parties into 6 ideological groups according to the left-right scale using the following intervals: far-left $0 < 1.5$, left $1.5 < 3.5$ centre-left $3.5 < 5$, centre-right $5 < 6.5$, right $6.5 < 8.5$, far-right $8.5 < 10$. In the analyzed period, the right parties group is the set with the highest number of posts per party on median (2088). In contrast, the left parties are clearly the group where parties published the least posts on median (1598), which represents an outlier in comparison with the other ideological groups. Finally, Appendix A.2 includes a figure with the number of posts published per week for each of the six ideological groups considered. All the ideologies present similar patterns with an increase in activity around electoral periods.

In a nutshell, our results reveal that parties on the right-wing spectrum are more active in FB than parties on the left wing.

## Methods

This section introduces the methodology we propose to predict the ideology and polarization of EU + UK countries using the FB dataset we have collected for this purpose. We organize the section as follows: (i) we briefly introduce the metrics of ideology and polarization used in this paper along with the ground truth data used to train our prediction model; (ii) we list the variables extracted from our FB dataset to be used as input of our model; (iii) we describe our regression model to predict the ideology and polarization metrics.

### Ideology and party system polarization definition

The political picture of a country can be defined in many different ways. Each country has its unique national issues and particularities that are intrinsic to them. However, our goal is to study and compare the European political framework to build an automatic and general predictor of the ideology and polarization that can be applied to all EU + UK countries. However, ideology and polarization are different metrics that require different definitions.

The first one, *country ideology*, specifies the general ideology of a country, which is known as the ideological center of gravity [40]. In other words, we compute the country ideology as the weighted average ideology according to the portion of votes received by each political party, as follows:

1 $$ \mathit{II}_{c} = \frac{\sum_{i=1}^{N}\mathit{VoteShare}_{i} * \mathit{PartyLR}_{i}}{\sum_{i=1}^{N}\mathit{VoteShare}_{i} }, $$

where *VoteShare* refers to the percentage of votes received by party i in country c, and *PartyLR* to the left-right value of that party. In this paper, the ideology of a party is defined as a value in the left-right scale between 0 (far-left ideology) and 10 (far-right ideology). We will use the ideological value ParlGov assigns EU + UK political parties.

The second one, *polarization*, measures the ideological spread of a particular country. There are multiple definitions of political polarization, in particular, in this paper, we use the Party System Polarization which measures the variance in the distribution of parties in a parliament in the left-right dimension. We can find multiple metrics to measure party polarization in the literature. Some authors propose to use the fragmentation of a party system with metrics such as the Herfindahl index or the Laakso and Taagepera index [41] (they are interchangeable since one is the inverse of the other). These indexes use the number of effective parties, reducing the relevance of small parties. We can find more sophisticated polarization indexes that consider the ideology of the parties on the left-right scale. For instance, Sigelman and Yough [42] proposed a metric introducing the ideology of each party. In this metric, the polarization is computed with a quadratic formulation that introduces relatively large polarization changes associated with small ideology variations. Dalton [43] defined an improved index that presented a more balanced scale that facilitates the comparison among countries and the interpretability of the results. Finally, other proposals combine both inputs, the number of parties and the ideology. An example is introduced in [44], where authors use an index similar to Dalton’s taking also into consideration the number of effective parties.

In this work, we choose the *Polarization Index (PI)* proposed by Dalton [43] since, compared to the state-of-the-art metrics, Dalton’s PI allows us to: $(i)$ do a cross-national comparison with a simple index, $(\mathit{ii})$ consider the parties relevance and their position in a political scale, $(\mathit{iii})$ it is bounded between 0 and 10 as the left-right scale, which simplifies the comparison and interpretability of the results. The *Polarization Index* proposed by Dalton is defined as:

2 $$ \mathit{PI}_{c} = \sqrt{\sum_{i=1}^{N} \mathit{VoteShare}_{i} * \biggl( \frac{\mathit{PartyLR}_{i}-\mathit{II}_{c}}{5} \biggr)^{2}}, $$

where $\mathit{II}_{c}$ represents the ideology value of the country *c* (see Equation (1)). Hence, this index allows us to understand the spread of polarization between a group of *N* analyzed parties. Particularly, a value of 0 means that every party is in the same ideological position, *i.e.*, the same left-right ideology value; and a value of 10 would mean that the vote is split in half in the opposite positions of the ideological spectrum.

### Ground truth

Before discussing how to estimate the ideology and polarization of a country over time, it is necessary to have a reliable ground-truth reference for the ideology and polarization metrics we have defined. The best proxy for our approach is the electoral results. This proxy assumes that the aggregate ideology of a country is linked to the percentage of votes received by the different political parties with different ideologies. Therefore, we use electoral results as ground truth for our model.

Since the main goal of our paper is to create an ideology and polarization prediction model that can be used in any of the 28 countries considered in our work, the best ground truth dataset is the results of the May 2019 EU parliament elections because all the 28 countries run elections in parallel. The EU parliament ballot results have been used as the ground-truth information to train our regression model.

We could have used instead, the results of the 28 countries’ national elections. Unfortunately, in the 3 years considered in our work, not all the countries had national elections. Nevertheless, we will use the results of 15 national elections between 2019 and 2020 to validate the performance of our ideology and polarization prediction models.

As we introduced above, to obtain the *country ideology* in a given country based on the actual ballot results, we compute a weighted mean taking into account the *Vote Share* of each party in the election along with their ideology value on the scale of 0-10. Analogously, we use the Dalton formula (see Equation (2)) to compute the *polarization* ground truth using the electoral results.

### List of Facebook variables considered in the prediction model

Based on the data extracted from Facebook, we can compute a country’s ideology and polarization by using different variables from our dataset. First, we calculate the value of a variable for every party in a country (e.g., the total number of posts published by each party), then, we express those variables as a percentage. Finally, we get an ideology or polarization value for each country using the formulas described in Sect. 3.1, replacing the Vote Share for the percentages calculated. Our dataset includes variables covering three dimensions: popularity, activity, and engagement. It is worth noting that we use those dimensions to replace the Vote Share as previous works have studied the relationship between them [26, 27, 32].

For popularity, we use the number of followers and page likes from political parties’ Facebook pages. To capture activity we rely on two metrics related to the posting activity of political parties: the total number of posts and the posts published per day, which measures the median time difference of two consecutive posts by a party, expressed as the number of publications made each day. Finally, to cover the engagement attracted by political parties we create multiple metrics extracted from the reactions, comments, shares, and video views received by political parties’ posts. In particular, if we consider reactions^2^ we produce the following metrics per political party: the total number of reactions from all the aggregated posts, the median number of reactions per post, the average number of reactions per post, and the median number of reactions per day or week. We compute the same metrics for comments, shares, and video views as well. We have decided to use multiple time windows (day, week, all-time) as well as the median and the mean in the case of engagement because we were not sure beforehand which metric would work better in the model.

Table 2 summarizes all the variables we have just introduced. These variables represent the set we will explore to create our prediction model, but the model, as we will see later, will not use all of them.

**Table 2:** List of metrics used to understand, build and compute the ideology and polarization regression model

total	median	mean	median day	median week
posts	posts_per_day			
reactions	reactions_median	reactions_mean	reactions_median_day	reactions_median_week
comments	comments_median	comments_mean	comments_median_day	comments_median_week
shares	shares_median	shares_mean	shares_median_day	shares_median_week
views	views_median	views_mean	views_median_day	views_median_week
page_likes				
followers				

The metrics are derived from the data from each post (comments, shares, reactions, video views) and the pages of political parties (number of followers, page likes).

The methodology to create the ideology and polarization models based on Facebook data and assess their quality is divided into three steps: (i) we first find correlations among the variables included in Table 2 to eliminate those variables highly correlated; (ii) second, we build and train multiple regression models using different combinations of variables and select the one showing the best performance based on the ground-truth data obtained from the May 2019 EU parliament elections. We also analyze the results using different data time windows to understand what is the most appropriate time span to maximize the performance of our model. For the month before the elections, we use a time window for every week, and the rest are every other week except the last one, *i.e.*, the largest time window covers 4 months and 3 weeks from Jan. 1 2019 until May 21. 2019, two days before the EU parliament elections started.^3^ The shortest time window is one week before the elections. (iii) We use the electoral results of national elections to assess the performance of our prediction model.

Finally, we note that we tried to use regularization techniques using the lasso regression [45], which led to similar results, but the performance did not improve overall. In Appendix A.6 we discuss the models and results obtained with Lasso regression.

### Regression model

We introduce the described methodology to build the best-performing regression model to estimate the ideology and polarization of a country using Facebook information from political parties. We first break down and study the correlation between the metrics presented in Sect. 3.3. Then, we display the procedure used to train the model. Finally, we depict the cross-validation methodology that allows for finding the most promising models among the tested ones.

#### Variables selection

A good variable selection is crucial to building an efficient model and preventing cases of over and underfitting. We study the correlation between every pair of metrics to understand which ones are equivalent and highly correlated. Hence, if we find several correlated metrics, we will only use one of them in the model.

**Figure 5:**
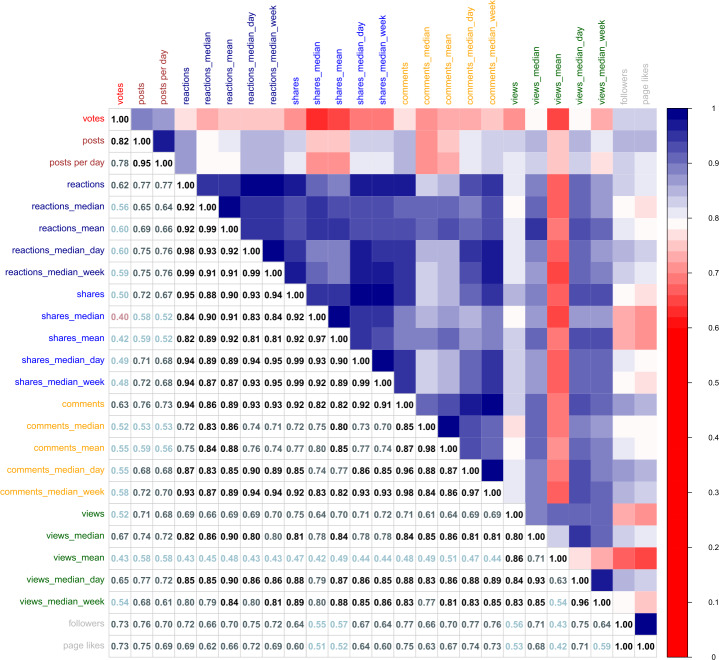
Pearson correlation between Country Ideology variables

Figure 5 shows the correlation between each pair of ideology metrics considering the whole data period from January 1, 2019, to December 31, 2021. The Figure displays both a heat color map (right upper part) and the values of the Pearson correlation (left lower part) between each pair of metrics in our study. The diagonal represents the correlation of a variable with itself, and therefore, it will always be 1. This analysis will allow unveiling which variables are highly correlated to avoid using them together in the model.

We find high correlations in the activity variables among them and the engagement variables among them above. In many cases, this correlation is above 0.85. Contrarily, the correlation is generally considerably lower when you compare variables belonging to different types. The max and min correlation between engagement and activity variables are 0.78 and 0.51, respectively. The popularity metrics are the ones showing less correlation among them and with the engagement and activity group. We note that when we consider a different time window the correlation values between pairs of metrics could change. To create our regression models we avoid using together variables that show a $\text{correlation} \geq 0.60$.

#### Multiple regression models

The number of samples to build our model is limited to the 27 countries in our research.^4^ For this reason, the regression analysis should not contain many independent variables. We consider a general rule of thumb for the sample size, where there should be at least 10 samples per model variable [46]. Hence, we set to 2 the number of metrics we can use for our model to be representative enough. There could be other metrics that may add value to the regression, however, due to the limited number of samples we avoid using more than 2 variables to prevent our model from overfitting. Therefore, we will construct our final linear regression model (both for ideology and polarization) as in Equation (3):

3 $$ y \sim \beta _{0} + \beta _{1} x_{1} + \beta _{2} x_{2} $$

Where *y* is the predicted ideology (or polarization) value, $x_{1}$ and $x_{2}$ are the computed values with the dataset, and *β* describes the coefficients.

We remind that we trained models using different time windows (as described in Sect. 3.3) for all pairs of metrics with a correlation lower than 0.60. It is worth noting that we eliminated the models where any of the variables have no statistical significance *i.e.*, *p-value* greater than 0.05 in any of the features or the intercept. Finally, we compute the Variance Inflation Factor (VIF) metric for all of the models ensuring they do not present any colinearity.

In order to discern which are the best models to estimate both the ideology and polarization, it is necessary to perform some kind of validation. For this purpose, we perform a *Repeated K-Fold Cross-Validation* using the data from the European Elections. This validation allows us to measure the success of our model and pick the ones achieving the greatest accuracy.

To increase the robustness of the cross-validation, we used the following procedure:

$(i)$
For each country in our dataset $c_{i}$ ($i \in [1,27]$), we include each $c_{i}$ in a random group $r_{k}$ ($k \in [1,5]$). Having 27 samples, it is not possible to split the data in a uniformly balanced manner, the majority of folds would include 5 samples but some of them would vary in size from 4 to 7 samples.
$(\mathit{ii})$
Following, to train and cross-validate the models of Sect. 3.4.2, we use $k-1$ (4 out of 5 in our case) groups to train the models and the remaining *k* to validate the result and compute the accuracy metrics coefficient of determination ($R^{2}$) and Root-Mean-Square Error (*RMSE*). We take all the combinations in this process and repeat the cross-validation *k* times. Therefore, first, we use $r_{k} \in [1,4]$ to train the model and $r_{5}$ to validate it. Second, we use $r_{k} \in 1,2,3,5$ to train the model and $r_{4}$ to validate. Again, we follow this procedure for all the $r_{k}$ combinations letting aside one $r_{k}$ for validation. Lastly, we extract the aggregated $R^{2}$ and *RMSE*.

To increase the robustness of the cross-validation, the process $(i)$ is iterated 100 times, and each time we include each $c_{i}$ in a random group $r_{k}$. After that, the K-Fold Cross-Validation method $(\mathit{ii})$ of the previous paragraph is repeated. Finally, we average the cross-validation results of those iterations, providing insight into how the models work in comparison with each other.

**Figure 6:**
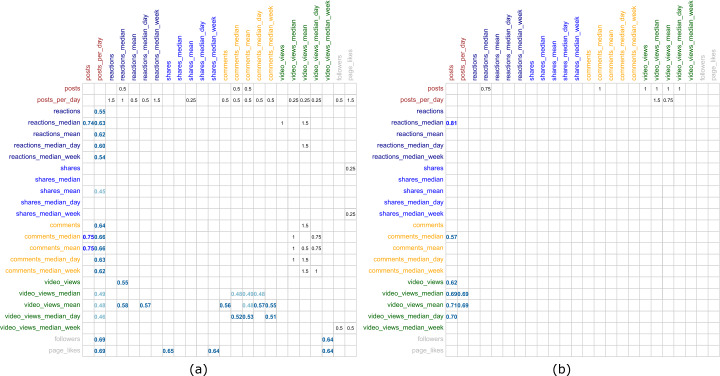
Cross-validated linear regression models. (**a**) Ideology models, (**b**) Polarization models

Figure 6 shows the R^2^ value for all the models cross-validated for the different combinations of variables. The figure shows in the left-side diagonal the maximum R^2^ value among the evaluated time windows. The right-side diagonal depicts the time window (in months before the elections) that maximizes the R^2^ for each pair of variables. In Table 3 a summary of the best performing models is shown, including variables used, the value of coefficients and their statistical significance, and the performance of the model (R^2^ & RMSE), and the optimal time window. In this table, we did not include pairs of variables of the same group, *i.e.*, $I_{2}$ (*posts* and *reactions_median*) is the best performing model using posts and reactions, therefore the model of *posts* and *reactions_mean* is not included.

**Table 3:** Best performing models

Model	Var. 1	Coef.	Var. 2	Coef.	Inter.	R^2^	RMSE	Months
$I_{1}$	posts	0.504(***)	comments_mean	0.123(*)	2.061(***)	0.752	0.287	0.5
$I_{2}$	posts	0.479(***)	reactions_median	0.148(*)	2.039(***)	0.735	0.292	0.5
$I_{3}$	posts_per_day	0.314(***)	page_likes	0.297(**)	2.069(***)	0.707	0.315	2
$I_{4}$	shares	0.161(*)	page_likes	0.324(**)	2.723(***)	0.652	0.354	0.25
$I_{5}$	video_views_median_week	0.198(*)	page_likes	0.329(**)	2.545(***)	0.649	0.351	0.75
$I_{6}$	posts_per_day	0.304(**)	video_views_median_week	0.220(*)	2.545(***)	0.602	0.366	3
$I_{7}$	reactions_mean	0.279(*)	video_views_mean	0.147(*)	3.125(***)	0.591	0.406	2
$I_{8}$	comments_median_week	0.184(*)	video_views_mean	0.183(**)	3.467(***)	0.576	0.416	2
$I_{9}$	posts_per_day	0.233(*)	shares_mean	0.185(*)	3.147(***)	0.448	0.463	0.25
$P_{1}$	posts	0.210(*)	reactions_median	0.546(***)	0.793(*)	0.808	0.424	0.75
$P_{2}$	posts	0.274(*)	video_views_mean	0.355(***)	1.46283(**)	0.708	0.531	1
$P_{3}$	posts	0.354(*)	comments_median	0.261(*)	1.526(**)	0.568	0.608	1

Signif. codes: 0 ‘***’ 0.001 ‘**’ 0.01 ‘*’ 0.05 ‘.’ 0.1 ‘ ’ 1.

For polarization models, it is clear that $P_{1}$ is by far the best model, as it provides a $R^{2}$ of 0.8, this model uses *posts* and *reactions_median* to estimate this metric. In the case of ideology, both $I_{1}$ and $I_{2}$ provide a very similar result in both R^2^ and RMSE, selecting one or another should not impact the performance. Therefore, we selected the $I_{1}$ model, as its RMSE is slightly lower.

#### Temporal analysis of the selected models

Even though the models had been cross-validated, it is necessary to explore how the performance of these models changes when the time window varies in size, as we would like our models to work in different window lengths. This analysis is relevant and necessary since our purpose is to depict a time window where our models are accurate in the case at some point we are not able to collect the optimum data period from Facebook pages.

**Figure 7:**
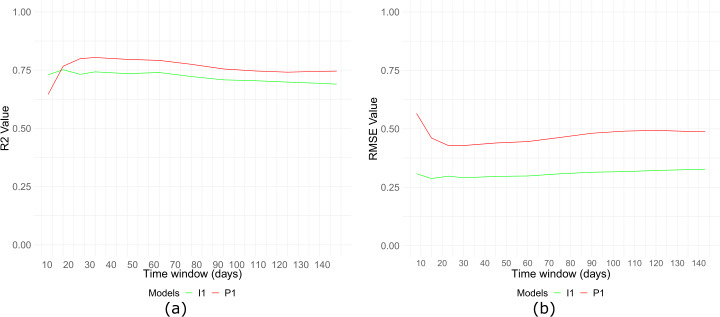
Selected models with different time windows. (**a**) R^2^ (**b**) RMSE

Figure 7 shows the performance of these models for time windows between 7 to almost 150 days, using the cross-validation methodology of the previous section.

On the one hand, for the case of the country’s ideology, the $I_{1}$ model displays the best performance when the time window is between 15 and 75 days. Particularly, $I_{1}$ provides the lowest RMSE (0.276) with a 0.5 months time window of data. Therefore this model shows a great estimation achievement in that range, with an RMSE lower than 0.31 and R^2^ greater than 0.72, making it also flexible in terms of data input. Outside of that range, $I_{1}$ falls to lower accuracy, although the performance would be still acceptable.

On the other hand, for the case of the country’s Dalton polarization, $P_{1}$ also performs better in time windows between 15 and 75 days, with an RMSE lower than 0.47 and R^2^ greater than 0.75.

Although the optimal time windows are quite short, we would like to clarify that models with wider time windows for both ideology and polarization also show similar performances, this suggests that the effects of electoral campaigns (such as political parties being more active or people engaging more with political posts) does not affect the performance of the methodology followed. If the models were only to work due to campaign effects, there would be visible a drop in performance in Fig. 7.

In summary, the analysis performed to get the best ideology and polarization estimations in a country by using Facebook data shows that models $I_{1}$ and $P_{1}$ behave great with a data time window between 15 and 75 days. These models use only 2 variables as input and their error is very small, the coefficients and the time windows selected are the ones that were shown in Table 3.

It is important to highlight that although we only used 2 variables, adding more features could improve the accuracy and diminish the error of estimations. However, we would need a higher sample size to provide meaningful insights in this term. To the best of the authors’ knowledge, this is the first study that estimates a country’s ideology and polarization with predictive models relying on Facebook data. In the following section, we explore the actual predicting ability of $I_{1}$ and $P_{1}$ in real Parliament Elections of 15 individual countries.

## Results

To evaluate the performance of the selected models, i.e., $I_{1}$ and $P_{1}$, we compare their ideology and polarization predictions to the actual ballot results from 15 individual national parliamentary elections that took place in 2019 and 2020. Even more, to further validate our model, we also use four elections happening in 2016 to understand whether the performance of our model is also good even to predict ideology and polarization in the past. If the results are good for both recent elections and past elections, this would mean our models may be a good tool for social scientists, political scientists, etc. to study EU ideology and polarization over long time periods, which would allow monitoring both political variables on a continuous basis, to observe changes in tendencies even between two close elections.

### Model performance evaluation using 15 recent individual country elections

For each of the elections happening in 2019 and 2020, we compute the Ideology and Polarization values as described in Sect. 3.1, using the Vote Share of each party with its left-right value as reported by ParlGov. Then, we apply models $I_{1}$ and $P_{1}$ to predict the ideology and polarization of the country running the election, and measure the error of our prediction.

Table 4 and Table 5 show the date of each election and the predicted and real values for country ideology and Dalton polarization together with the error of the predictions. The tables also show the baseline model performance, which uses the previous known ideology and polarization point, *i.e.*, using the results of the previous European election, to test whether the past information is an accurate indicator of either metric. Finally, the last row of the table assesses the model accuracy using the RMSE across the 15 elections under analysis. Overall the results are surprisingly good with an average RMSE of 0.28 for ideology and 0.27 for polarization. That means, our models are properly capturing the global ideology and polarization of a country according to the actual ballot results in most cases.

**Table 4:** Ideology Prediction results in 15 individual country elections

Country	Date	Real	BL pred	BL Error	$I_{1}$ pred	$I_{1}$ error
Austria	2019-09-29	5.512	5.487	0.025	5.417	0.096
Belgium	2019-05-26	5.324	5.353	−0.028	5.521	0.196
Croatia	2020-07-05	5.894	5.512	0.382	5.635	0.259
Denmark	2019-06-05	5.260	4.970	0.290	5.172	0.089
Estonia	2019-03-03	5.842	6.105	−0.263	5.233	0.609
Finland	2019-04-14	5.353	5.530	−0.176	5.759	−0.405
Greece	2019-07-07	4.781	4.978	−0.197	4.752	0.029
Ireland	2020-02-08	4.583	4.548	0.035	4.690	−0.107
Lithuania	2020-10-11	5.283	5.134	0.149	4.982	0.301
Poland	2019-10-13	6.351	6.837	−0.486	6.233	0.119
Portugal	2019-10-06	4.580	4.507	0.073	4.306	0.274
Romania	2020-12-06	5.441	5.362	0.078	5.792	−0.352
Slovakia	2020-02-29	6.465	6.335	0.130	6.104	0.360
Spain	2019-11-10	5.271	5.103	0.168	4.963	0.308
UK	2019-12-12	5.722	5.940	−0.219	5.802	−0.080
			Baseline model		$I_{1}$ model	
RMSE			0.221		0.284

**Table 5:** Polarization Prediction results in 15 individual country elections

Country	Date	Real	BL pred	BL Error	$P_{1}$ pred	$P_{1}$ error
Austria	2019-09-29	3.928	3.959	−0.031	3.986	−0.058
Belgium	2019-05-26	5.005	4.130	0.875	4.670	0.335
Croatia	2020-07-05	4.385	4.166	0.219	4.503	−0.118
Denmark	2019-06-05	4.666	4.727	−0.061	4.753	−0.087
Estonia	2019-03-03	3.886	4.071	−0.185	3.432	0.454
Finland	2019-04-14	3.485	3.426	0.059	3.683	−0.198
Greece	2019-07-07	4.371	4.625	−0.253	4.070	0.302
Ireland	2020-02-08	3.645	4.036	−0.391	3.371	0.274
Lithuania	2020-10-11	3.938	4.192	−0.254	4.096	−0.158
Poland	2019-10-13	3.383	2.495	0.888	3.437	−0.054
Portugal	2019-10-06	3.627	3.825	−0.197	3.488	0.124
Romania	2020-12-06	3.364	2.401	0.963	3.491	−0.127
Slovakia	2020-02-29	3.720	3.278	0.442	3.591	0.129
Spain	2019-11-10	5.195	4.504	0.690	5.409	−0.214
UK	2019-12-12	3.447	4.817	−1.369	4.106	0.658
			Baseline model		$P_{1}$ model	
RMSE			0.602		0.270	

If we look at the results for political ideology, we observe that our models are quite accurate in most of the cases since the error for $2/5$ and $3/5$ of the countries is lower than 0.15 and 0.3, respectively. The higher error happens in Estonia. However, we believe the error is still acceptable since it is slightly deviating to the right from the actual ideology shown by the electoral results. Even though is worth mentioning that the baseline obtains slightly better results, meaning that the ideology of a country stays rather static between two consecutive elections.

We observe similar results for the case of polarization where $7/15$ countries show an error lower than 0.15 and $11/15$ lower than 0.3. The worst prediction happens in the UK where our model overestimates the polarization with an error of 0.658. However, the results of the $P_{1}$ are highly better than the baseline model. In this case, the baseline model is not enough to predict the polarization changes.

Overall, the obtained results demonstrate that our models can be used as accurate proxies to monitor political ideology and polarization in Europe. This means that the information embedded in the activity of political parties on their FB pages can be exploited to produce real-time monitoring of the ideology and polarization evolution in Europe.

### Performance results in prior elections

We have demonstrated that our model is a good proxy to predict political ideology and polarization in a 2 years period. However, we believe our models would be a much more useful tool to provide ideology and polarization metrics if we could prove they are also efficient to predict both metrics prior to the period analyzed. This means social scientists, political scientists, etc. could use the ideology and polarization metrics resulting from our model over long periods of time for their studies.

To carry out this analysis we had to extend our data collection beyond the initial planned period, i.e., January 1, 2019, to December 31, 2021. We selected four countries running elections in 2016: Ireland, Lithuania, Slovakia, and Spain; and one from 2017: Germany and collected the information from the FB pages of their political parties a few months before the elections. We then used $I_{1}$ and $P_{1}$ and data prior (0.5 and 0.75 months of prior data) to each election to predict the ideology and polarization in each of the 5 compared countries, and compare the model predictions to the actual ideology and polarization extracted from the ballot results. Table 6 shows country ideology and Dalton polarization resulting from our model predictions and the election results along with the associated error. At the bottom of the table, we show the RMSE for the four elections under analysis.

**Table 6:** Prediction results in 5 country elections from 2016 and 2017

Country	Date	Country Ideology			Dalton Polarization		
		Real	Pred	Error	Real	Pred	Error
Germany	2017-09-24	5.280	5.738	−0.458	4.433	5.000	−0.567
Ireland	2016-02-26	4.955	4.927	0.028	3.618	3.226	0.392
Lithuania	2016-10-23	5.261	5.434	−0.173	4.237	3.904	0.328
Slovakia	2016-03-05	6.164	6.379	−0.215	3.919	3.849	0.071
Spain	2016-06-26	4.935	5.120	−0.184	4.930	4.264	0.667
		Country Ideology			Dalton Polarization		
RMSE		0.253			0.454		

We observe that the performance of our political ideology model (RMSE = 0.253) obtains slightly better results in these five elections prior (2016, 2017) to the EU parliament elections and the 15 elections run between 2019 and 2020. In the case of the Dalton polarization our model shows a slightly worse performance when considering the 5 elections RMSE = 0.454). Still, we believe it is a rather good estimator to measure the polarization of EU countries in the past if we consider that the Dalton polarization ranges between 0 and 10.

## EU Political Barometer website

To complete our research work we have built an interactive website, referred to as *EU Political Barometer*, that allows other researchers, stakeholders, and any other interested person, to easily get access to our research results. Our website displays information about the activity and engagement per political party and country over time as well as the evolution of the political ideology and polarization of the EU + UK countries based on the predictions extracted from our models.

Our website displays the information from Jan. 1st, 2019 until the present day since we keep collecting the public posts from the political parties’ FB pages continuously. That means, our website will show up-to-date information about activity, engagement, ideology, and polarization as well as historical information from Jan. 2019.

We believe our *EU Political Barometer* represents a valuable contribution to the EU landscape since to the best of our knowledge is the first effort of this nature that aggregates together relevant insights of political activity across the 27 EU member states plus the UK. Even more, we aim to contribute to the open science by making openly downloadable the estimations of our model for ideology and polarization for each of the 28 countries considered in a standard format such as CSV files. This way we aim that anyone willing to use our predictions of ideology and polarization does not need to replicate our whole methodology and especially avoids collecting all the required data. We believe this represents a positive and relevant contribution to the open science effort. The *EU Political Barometer* is publicly available at https://eupoliticalbarometer.uc3m.es.

Next, we briefly describe a few dashboards available on our website that directly relates to the content of this paper. It is important to note that the website includes other elements beyond what we cover in this paper.

**Figure 8:**
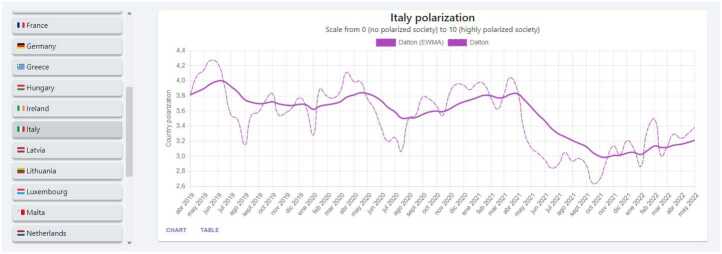
Dalton Polarization in Italy

**Figure 9:**
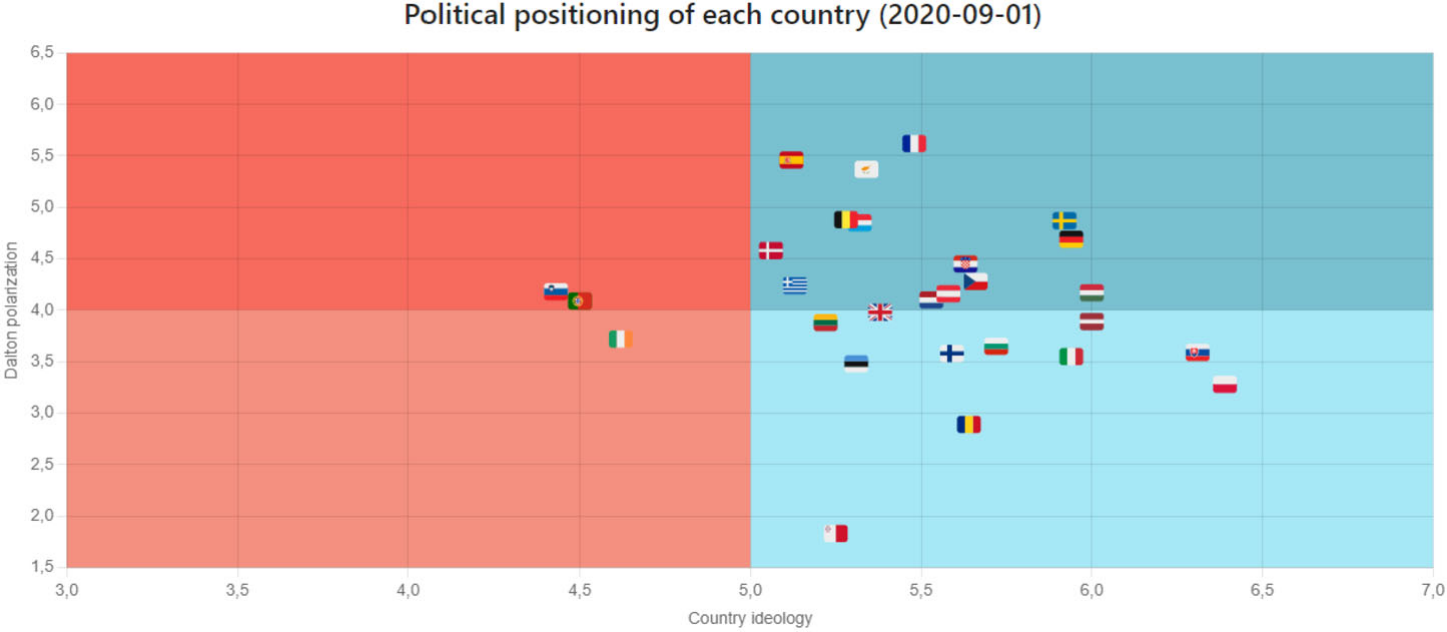
Political positions of European Countries

**Figure 10:**
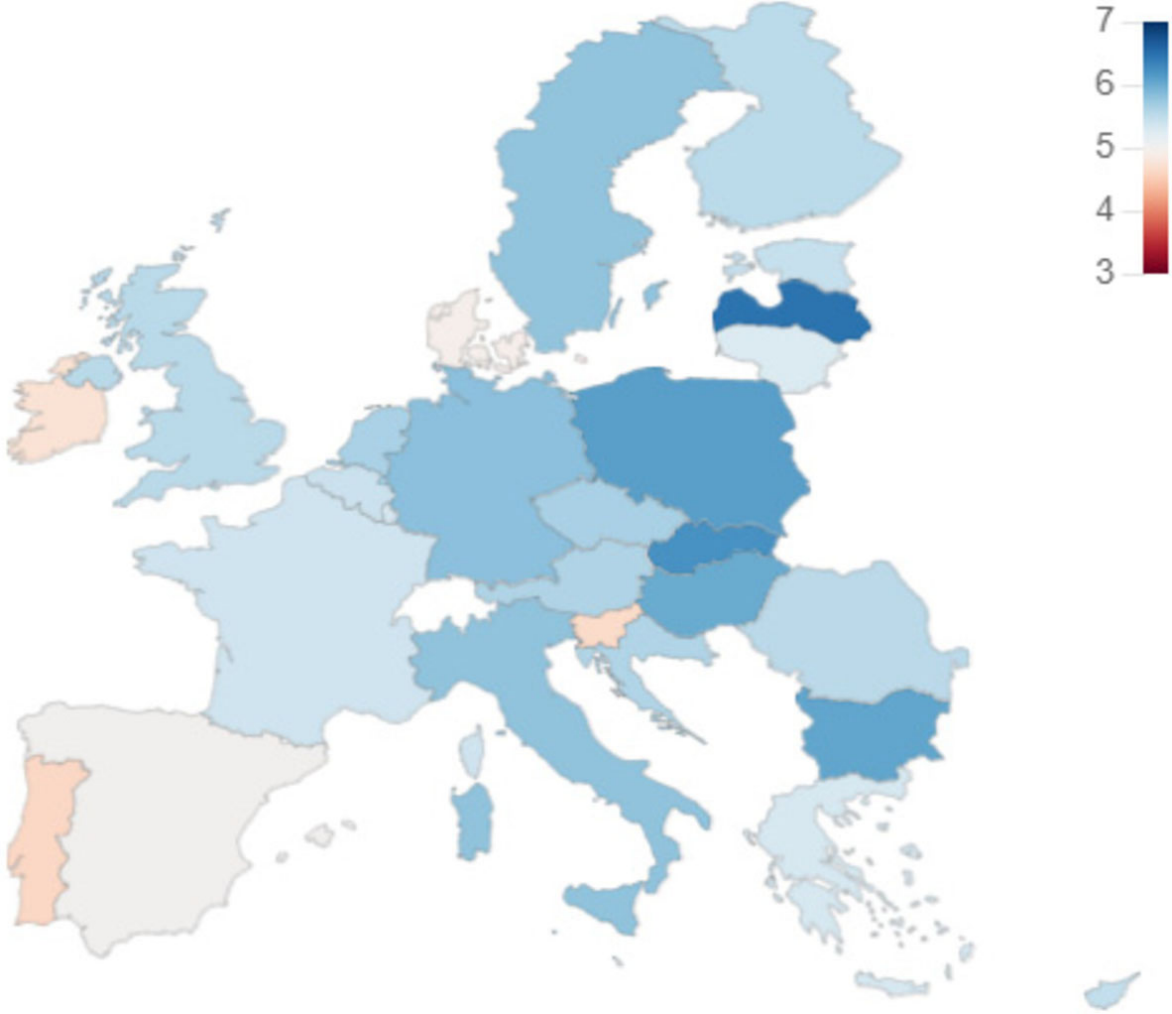
Political Ideology of European Countries
- *Dashboard per country/party*: We have a specific dashboard that allows the user to select any country or party they are interested in. For the selected country/party the dashboard displays multiple panels depicting (among other things): ideology, polarization, activity, and engagement over time. The user can zoom in to look into detail at a specific time window. In the case of countries, the figures represent the aggregated results across all political parties. As an illustrative example, Fig. 11 shows (for the case of Austria) one of the panels included in the website that represents the portion of posts contributed by each political party over time.

- *Ideology & Polarization*: In this dashboard, the user can go through several panels covering the ideology and polarization of a country. The models resulting from our research allow us to estimate the ideology and polarization metrics of a country at any point in time. In addition, the flexibility of our solution allows using different time resolutions to visualize the results. Figure 8 shows an example of the Ideology & Polarization panel, where we observe the evolution of Italy’s polarization across different months. The figure includes two lines, one for the predicted values, and the other for the EWMA (Exponentially Weighted Moving Average), as is necessary to consider the small sizes of the time windows of the selected models. This dashboard includes another very interesting panel (Fig. 9) where we plot in a two-dimension plane, where the *x*-axis refers to ideology and the *y*-axis to polarization, the results for each country denoted using their flag. Users can use a time bar to visualize the results at a specific time or can play a video that shows how the ideology and polarization of each country evolve over time. We also provide a panel that includes choropleths showing the ideology and polarization in maps of Europe (see Fig. 10).

**Figure 11:**
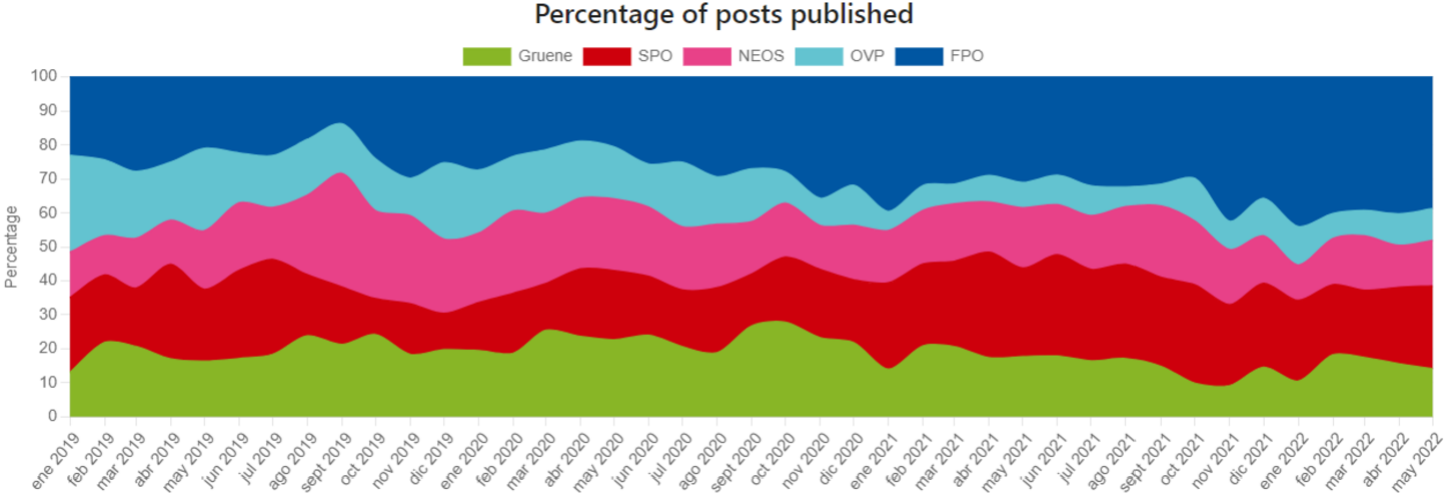
Percentage of pots published by each party in Austria
  In general, the feature of breaking down the data per country/party provides the user with much practical information about the political landscape of that particular country/party.
- *Comparison dashboard*: We developed a specific dashboard for comparing countries and/or political parties. This is a very interesting functionality since it allows comparing in a single figure the difference (over time) among two or more countries/parties across different metrics. As an illustrative example, Fig. 12 shows the selection panel where we have choose three far-right parties in Europe: *Rassemblement National* (France), *Vox* (Spain), *Alternative für Deutschland* (Germany). In turn, Fig. 13 shows the website panel comparing the activity (posts per week) for those three political parties.

**Figure 12:**
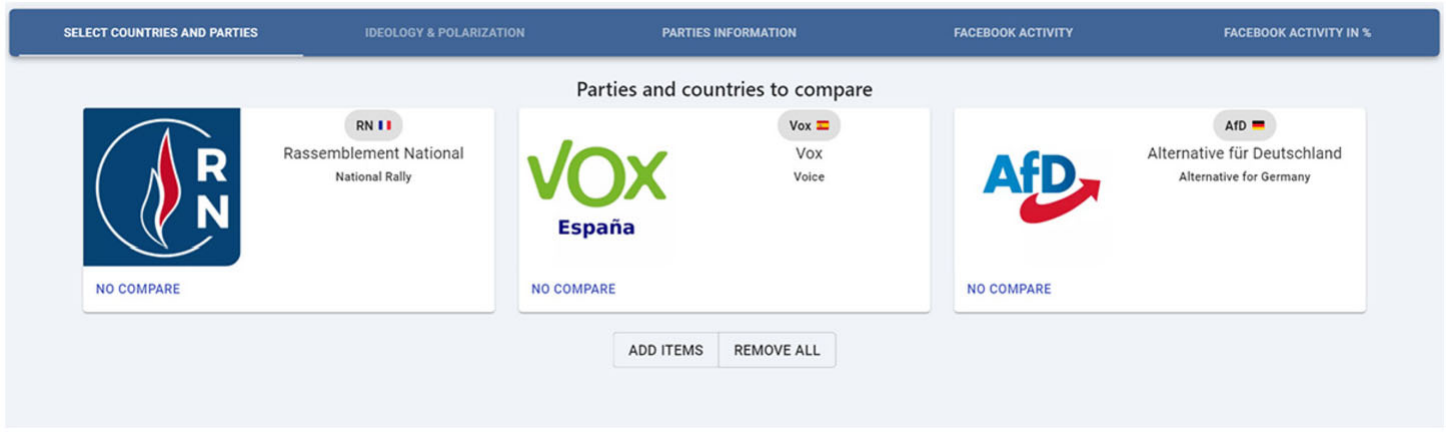
Selection of parties to compare

**Figure 13:**
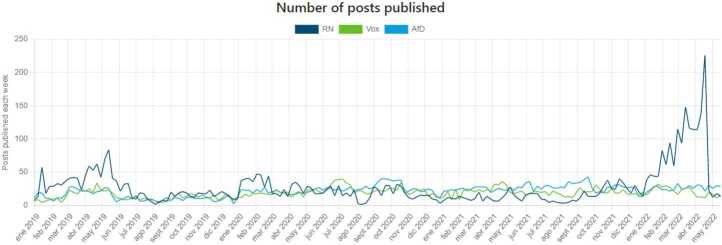
Post-activity of RN, Vox and AfD
- *Country stats dashboard*: We also included a dashboard where the user can see the statistics and information about our dataset. It displays the number of posts, reactions, parties, followers, and other parameters related to the dataset of a particular country, and also the aggregated values of all countries.

To sum up, this section has introduced another major outcome of our research, the EU Political Barometer. This website leverages the output from the methodology described in this paper to obtain the estimations of the political ideology and polarization, which represent one of the most relevant insights of the barometer.

## Discussion

Our results suggest that the ideology of a country can be predicted through a simple model that combines activity and engagement from political parties’ pages. However, it is important to acknowledge that scientifically explaining the causal relationship embedded in any model using social media to predict political outcomes such as ideology, polarization, vote share, etc, is a very complex task that requires a complete research work that goes beyond the scope of this paper. Although we cannot provide a scientifically proven causal link between activity, engagement and ideology, we believe it is important to include a speculative discussion to describe the complex elements embedded in a social media platform such as Facebook and roughly explain how they may be linked to the observed correlation in our results.

### Social media importance in political parties communication

There is a consensus that in the current society social media is an essential channel in political parties communication and more and more political actors use social media channels to directly convey their messages to the citizens [47, 48]. Among other things, social media allows political actors to communicate directly with citizens, circumventing the influence of traditional news outlets that could potentially shape the content of the messages they would like to project [49]. In addition, a recent report from Reuters on Digital News [50] indicates that global news interest has declined from 63% in 2017 to 51% in 2022. This decline has not been equally distributed across channels. The report indicates that social media news consumption is stagnant while traditional media news consumption is declining. Overall, we can safely state that social media platforms have become an essential part of citizens’ news consumption [47, 50].

Under the described scenario, it seems important for parties to be active in social media so they can convey their messages to the citizens as well as define strategies that allow them to increase as much as possible the engagement they attract through that activity. However, to the best of the authors knowledge no one has a master recipe to guarantee a successful strategy.

### Strategy and newsfeed algorithms

The participation of political parties on social media has the final objective of impacting the vote share results in elections. Which could be accomplished with different electoral goals, such as mobilizing supporters or persuading non-supporters [51]. Any of these strategies have a common ground in order to be successful, posts have to reach as many users as possible in order to achieve an efficient outcome. In other words, political parties aim is that the posts they publish become viral posts and reach a large number of users. Parties can follow different strategies to try to have a high impact on social media. However, the virality of a post is a very complex phenomenon that depends on multiple factors: social media platform algorithms, users proactively sharing the post, etc. Although parties are responsible for some aspects to achieve such virality, there is a fundamental factor that is out of control of the political organizations such as the social media platform algorithms.

These algorithms are specialized to deliver content with the objective to retain the user in the platform as much time as possible. This implies that the selection of posts is personalized for each user, which is done by using existing data of the user that the site had already collected. This means that political parties’ posts are competing with all kinds of content: influencers, celebrities, sport stars, news outlets, companies, etc. To the best of our knowledge there is no political party able to reverse engineering the algorithms in order to exploit them in their favor. Therefore, a big part of the engagement success of a post is out of the control of political parties.

Social media algorithms, including Facebook ones, are opaque and very complex. They combine many different factors to decide what posts may be relevant for a given user. The referred factors are used to compute a score for every possible post that can be delivered to a specific user. The posts with the highest scores will be displayed in the user’s newsfeed. Facebook vaguely clarifies how their algorithm works and unveils some of the factors their algorithms take into account [52, 53]. Some relevant factors are: (i) how often the user interacts with the posts of the account, group, or page, (ii) the popularity / engagement of a post: a post is more likely to appear if it has a high number of comments, reactions, or shares, (iii) how the user interacts with the same type of post (there are different types, such as text, image, video, link posts), (iv) how recently the content was posted. Finally, according to the available information Facebook avoids showing similar items to a user in a row. This means it is unlikely that a user receives two consecutive posts in their newsfeed from the same account.

Although it is clear algorithms are out of the control of political parties, they still have some room in order to maximize the chances to impact as many users as possible with their posts. In particular, based on the factors driving Facebook algorithm, political parties are responsible for: (i) increasing their user base, (ii) manage their posting frequency (i.e., activity), (iii) creating interesting content to increase the interaction of the users with their posts (i.e., engagement). We next briefly discuss each of these three elements.

#### Fanbase

**Figure 14:**
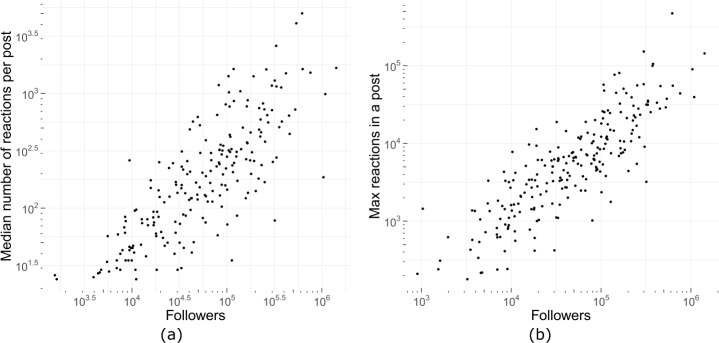
Number of followers and reactions by each party. (**a**) Median reactions per post (**b**) Maximum amount of reactions in a post

The fanbase of a political party is important to increase the impact of posts, as the number of followers is a proxy of the interest of users in a particular political party. Having more followers increases the chances of having your post delivered to more user feeds, which in turn will very likely improve your engagement. We validate this hypothesis using our dataset. Figure 14 represents the number of followers (*x* axis) Vs. the median number of reactions per post and the maximum number of reactions achieved by each party in their most viral post (*y* axis) on a logarithmic scale. The results show that the engagement of a political post is highly correlated to the number of followers of the political party on Facebook.

#### Activity

The political actors have control over their posting activity on the platform, as they can decide how frequently they post content on social media. There are two factors considered by Facebook posts selection algorithm that are relevant to make a decision: (i) Facebook algorithm will very rarely deliver consecutive posts from the same account to a user, (ii) Facebook will privilege recent posts over old ones.

On the one hand, publishing a post every few minutes does not make sense, as the newsfeed system will not promote the majority of posts to the pages’ followers. Therefore, a very high posting activity will lower attention per post. This effect can be seen near elections, as parties are more likely to increase their post rate, and we have observed that even though the total engagement increases, the attention per post is much lower. In addition, this effect can also be observed in individual parties like in the Lega per Salvini Premier in Italy, which is the most active party in Europe (and the most followed Italian party). However, their average engagement per post is lower than some other parties with a much smaller fanbase in Italy. They are the third and fifth party in terms of mean comments per post and median reactions per post respectively out of the seven Italian parties. Said that, their total engagement is much higher than the other parties.

On the other hand, having a very low posting rate (e.g., one post per week) is also inefficient since Facebook newsfeed algorithm privileges recent posts.

In a nutshell, political parties should apply a reasonable tradeoff and avoid very high or very low posting patterns.

#### Content of interest

Another aspect which is very relevant to the success of a post is the content itself. The more interesting the content for the users the more likely it is to obtain a large engagement. If users start liking, sharing, commenting, etc. on a post the algorithms will understand it is a relevant post and will very likely start delivering it more frequently in other users’ newsfeed. In other words, posts including content of high interests are much more likely to generate more engagement.

In a nutshell, while political parties can implement actions in order to improve the likelihood to increase engagement, they are still subject to the social media algorithms that ultimately decide which posts are delivered to which users.

### Filter bubbles

As we have discussed before, posting a high amount of content is not beneficial to the average amount of engagement, even though it is possible that the feed system can produce filter bubbles, which is a term that denotes the limitation of information that a user receives on a certain topic [54]. A user can fall in a political information bubble if their friends share a big proportion of posts from a particular side of the political spectrum, even with low engagement these posts can still be seen in the feed and have an impact on the user. This exposure to this type of content, which in some cases may include news, could then affect the user’s vote choice in elections towards that side [55]. On a related note, posts with news articles shared by friends are seen as more trustworthy than if the same article was received by the news outlet [56], reinforcing the idea of the formation of a political bubble or echo chamber.

### Limitations

Performing a comprehensive analysis of the impact of posts would require knowing how many times a given post has been displayed in users’ feeds. Borrowing the online advertising concept, the number of impressions of a post. The literature of online advertising has demonstrated that the more times you impact a user the more likely it is to influence them [57]. If we extrapolate that to political communication it would mean that showing your posts frequently to a given user may increase the possibility of influencing them. Therefore, a complete picture of the success of political parties posts in Facebook would require knowing the number of impressions. Unfortunately, this is something that only Facebook can do. An external researcher cannot obtain that information because it would require monitoring in real-time the posts displayed in all the newsfeeds.

Another metric that could also help to obtain a better understanding on this topic is the number of unique accounts interacting with parties’ posts, i.e., whether the engagement of the posts of a party always come from the same set of users or it varies depending on the content. If the posts of a party are always shared by the same people, their impact may be much lower that it seems.

In this paper, we have used instead the concept of engagement understood as the number of interactions of users with the political parties posts, which is something we can collect from Facebook. Therefore, our model uses the engagement as a proxy of posts success, which we believe is something reasonable but not complete.

### Summary

Our model suggests that high activity and high average engagement per post (i.e., mean comments for ideology and median reactions for polarization) leads to a higher vote rate that ultimately implies higher influence of that party in the ideology or polarization of a given country. Someone could then assume that based on our model if a political party suddenly increases their activity on Facebook, it would imply that in the short/medium term it will obtain better electoral results and would have more ideological influence in the country. However, it is important to clarify that as our results show that a higher activity correlates with a lower engagement per post. This means that increasing the activity negatively affects the engagement variable included in our models.

Therefore, what our model actually suggests is that the success of political parties requires a trade-off between activity and engagement per post. While political parties can manage the former part of the equation they cannot control the latter part, which very much depends on the social media algorithms as discussed before.

In summary, the lesson we extract from our results is that: (i) it is important that political parties are active in social media up to some extent. It is better publishing four posts per day than one post per month, in order to increase your engagement, but this is not enough; (ii) political parties need to create interesting posts that capture the attention of the users to increase the interaction with them and maximize the likelihood that social media algorithm understand this is a relevant post and display it more frequently in feeds; (iii) finally, in this same line, it is important that political parties implement actions to increase the number of followers, page likes, etc. which again may help to increase the engagement.

## Related work

There is a vast literature covering political ideology and polarization. We will organize the related work into subsections to better describe the different works relevant to our paper.

### Traditional methods

The majority of studies about political ideology and polarization rely on the traditional methodology of conducting surveys and evaluating the responses of interviewees. As stated in Sect. 1, researchers have used this methodology to analyze different socioeconomic parameters associated to political ideology and polarization [1–6, 8–10, 12, 15, 17, 19, 20].

Researchers have also tried to infer the political ideology of users and locations from surveys and polls. For instance, Wright et al. [58] collect several polls run by CBS News and the New York Times to create a base of 76k respondents to measure the ideology of US states. Even in that research, the authors acknowledge the need for massive data collection to achieve better results. For this reason, they propose to merge several polls’ information. More recently, Chirumbolo et al. [59] tried to estimate the ideology of 517 individuals who answered questions about their personality, ideology, and voting information. They then associate personality with different kinds of political ideology.

Although surveys are a very powerful tool to perform ideology/polarization analysis in well-defined and bounded human groups (e.g., country, region, etc.), they have inherent limitations due to the complexity, time-consumption, and cost of widely expanding them when for instance someone wants to cover many different countries. That is why there are only a limited number of efforts such as the World Value Survey [23] covering a large number of countries based on surveys. Our work contributes a methodology that infers political ideology and polarization at a very low-cost, low complexity, and high time granularity across many countries, which has been proven accurate.

### Social media

For a long time conducting surveys was the only way to measure the opinions and behaviors of users. Social media has become a powerful academic research tool. It avoids some of the limitations of traditional research: manual data entry, high costs, and time-consuming [60]. Extracting information from social media could also provide a broader spectrum of data that is not feasible to achieve with conventional surveys. Therefore, social media data have been used for multiple purposes related to our work.

#### Electoral results and popularity

We can find many works in the literature using social media data to predict electoral results. Most of these works rely on Twitter data. The work of Tumasjan et al. [61] is the first study to use Twitter to predict electoral results. They computed the number of tweets mentioning each party to estimate the 2009 German federal results. However, this research attracted criticism [62–64] upon issues such as the data collection procedure or the time window selected for their analysis. Another study used sentiment analysis [65] to show how tweets correlate to traditional political polls. Zohu et al. [66] used a dataset of 110M tweets from 6.3M users and build several AI models to predict the outcome of the 2019 Argentine elections. Aurav et al. [67] and Bermingham et al. [68] also use the volume of tweets mentioning a candidate before elections to estimate the outcome. Besides, DiGrazia et al. [69] found a high correlation between the number of tweets referencing a politician and their election results.

Researchers have also used Twitter to perform a sentiment analysis on political questions. There are few works [70–72] that have rely on sentiment analysis to estimate the number of seats in the 2015 Delhi, 2015 UK, and 2017 Punjab elections, respectively. Similarly, the sentiment analysis on Twitter have been used to predict the results for the 2012 US [73], 2016 US [74], 2018 Chilean [75], 2019 Indonesian [76], and 2020 US [77] presidential elections.

Beyond the prediction of electoral results, there are works [78] where the authors try to understand the popularity of political candidates in the Italian 2011 and French 2012 elections.

Although most of the works around electoral results are based on Twitter we can find few efforts using other platforms. For instance, in [79, 80] the authors used the volume of online searches using Google Trends to predict the outcome of US elections.

On Facebook, the relationship between the variables chosen in this article (*i.e.*, posting activity, post engagement, and popularity) and the vote share of a party is a topic that has been studied in the past, *e.g.*, Triantafillidou and Lappas [26] studied the 2019 general Greek elections in which the votes received was significantly related to different engagement measures (reactions, shares, and comments). Other studies had studied whether the followers of Facebook pages are related to the vote share obtained in elections, Cameron et al. [27] studied this phenomenon and they found a significant relationship between these variables, even though the effect seems to be small. The posting activity of politicians and its effect on vote share was studied by Hsin-Chen Lin [32], even though it seems that the number of posts in politician accounts is not significantly related to the vote share.

Our work differs from previous ones in two facts. First, we differ from them on the goal since we do not aim to predict electoral results but to obtain the ideology and polarization of a country. Second, all the introduced works focus on a single country whereas we are more ambitious and try to create a model valid across many countries.

#### Political ideology and polarization

More related to our work, social media has been also used to study the ideology of individuals and political messages. We can find works using both Twitter and Facebook data for this purpose

Barberá et al. [81] used Twitter data to analyze the communication and engagement of users to individuals with similar ideologies. Preoţiuc-Pietro et al. [82] relied on Twitter data to examine whether a user could be classified in a *left-right* scale by their posts published on Twitter.

Using Facebook data, Cecobelli [38] analyses the difference in the messages of political leaders from 18 countries. The results show that they tend to increase their post frequency and post content in the electoral campaign period. This is aligned with our activity analysis results presented in Sect. 2.2.1.

Ernst et al. [83] use data from Facebook and Twitter (845 Facebook posts and 555 tweets) from 88 politicians in 8 different countries. They found that populist ideas tend to compose the political strategy of politicians in the extremes of the left-right spectrum, more often than centrist politicians deliver that kind of message.

Bond et al. [84] collected data from 6.2M individuals who liked two or more verified Facebook pages from politicians. They build a model measuring the ratio of likes and fans on politicians’ pages and estimate the ideology. They provide a score of each politician’s page and estimate users’ ideology by considering an average of this score. Later, they validate the model against two sources: 20k+ users who disclosed their political ideology on their Facebook profile, and a survey conducted by over 20k+ users of their dataset, asking them to classify themselves in 5 ideological points. Finally, among other questions they explore, they find that women tend to be more liberal, and married couples correlate best in ideology.

Finally, Praet et al. use Facebook “likes” on pages as a tool to evaluate the political ideology of individuals, as likes on non-political Facebook pages could lead to identifying the ideology of a person [85], and also to study whether political polarization extends to lifestyle preferences [86].

Overall, our work contributes to the literature by proposing a new methodology to measure political ideology and polarization using Facebook data. In contrast to some of the works introduced above, we are not interested in measuring the ideology and polarization of an individual but in a country. Our approach allows the analysis of ideology and polarization indicators in a fine-grain time-continuous manner since we benefit from using information publicly available to anyone in any country. Moreover, our model focuses on a broad list of 28 countries (EU + UK), in contrast with previous works which focus on one or a reduced number of countries. We provide an easy way of doing cross-national analysis since the data necessary to estimate these measures is language-independent and only relies on posts’ indicators. The outcome models, both for ideology and polarization, work together in any of the 28 countries at any point in time. To the best of our knowledge, this is the broadest modeling of political ideology and polarization in terms of countries using social media data. As discussed in Sect. 4 the estimations are accurate to give an insight into the political situation and the evolution of each country.

## Conclusions

This paper leverages the public posts political parties publish on their main Facebook pages to estimate the political ideology and polarization across 28 countries, the 27 EU member states, and the UK. To this end, we have created a model for ideology and a model for polarization based on the activity of the political parties, the engagement they achieve with their posts, and the popularity of their Facebook pages. We have used the EU parliament elections held in May 2019, where all the 28 considered countries were involved, to build our model. We validated the accuracy of our model using 19 elections, 15 happening in the years 2019 and 2020, and the remaining 4 in 2016. The results show a very good performance of the proposed models leading to rather small errors compared to the ground truth derived from the actual ballot results. To the best of our knowledge, there is no prior work in the literature offering a solution that measures the ideology of all EU countries + UK countries.

Our methodology provides a few advantages over traditional methods meeting the requirements pursued in the design of this research: (i) it is a low-cost option. (ii) It allows measuring ideology/polarization at a high-resolution time scale. (iii) It works across 28 countries. Using traditional methods such as surveys, interviews, or polls to measure the ideology/polarization across 28 countries at a high time resolution (e.g., every week) is an unfeasible option due to its complexity and cost.

Therefore, our work delivers a novel tool that uses social media information to produce specific country metrics that can be used for further purposes. For instance, political ideology and polarization can be used by political scientists, social scientists, economic researchers, etc. as another metric in their studies to correlate ideology/polarization to other socio-economic metrics. In addition, our models have the advantage that they can estimate the ideology and polarization of a country as long as we can get access to roughly one month of public posts from the political parties’ FB pages within that country. This brings two important benefits: (i) we can monitor the evolution of the ideology/polarization at high time resolution (e.g., one week), which allows understanding tendencies in the ideology/polarization over pre-defined periods and may allow inferring whether specific events in a country are impacting on any of the two variables; (ii) since the public posts in FB pages of political parties are typically available from several years ago, our model allows obtaining estimations of the political ideology/polarization in the past. In many cases, researchers are interested in studying the impact of specific past events, and our methodology may be useful in this case as well.

Although our methodology is superior to traditional surveys for some features, we want to acknowledge upfront that traditional methods to measure ideology and polarization are still very relevant. Surveys are very useful to perform deep analysis over specific aspects related to ideology/polarization compared to our methodology. In summary, our research does not aim to invalidate traditional methods, but to coexist with them, and offer a new tool to the research community that may be useful for specific analyses that cannot be covered by surveys.

Moreover, we wanted to make the results of our work accessible and available to the research community, relevant stakeholders in the EU, and general individuals who may be interested in politics. To this end, we have built an interactive website referred to as *EU Political Barometer*. The site can be found at the following address: https://eupoliticalbarometer.uc3m.es. This website includes very detailed information about the activity and engagement of the 234 political parties in our dataset as well as the aggregated information per country from Jan 1, 2019, until the present. In addition, we also include the temporal evolution of our model estimations for the political ideology and polarization per country in the same period. Users accessing our website can easily select the country or political party of their interest and play with the temporal resolution they want to visualize. Therefore, one of the main advantages of this site is the possibility of displaying up-to-date historical data with a very small resolution span of one week. The results associated with multiple political parties or multiple countries can be visualized together to easily visualize differences among them. The website offers more information and functionalities that go beyond the scope of this paper. Our ultimate goal is to contribute to the open science initiative and allow other researchers to use our ideology and polarization estimations for their work.

## Figures and Tables

**Figure 15:**
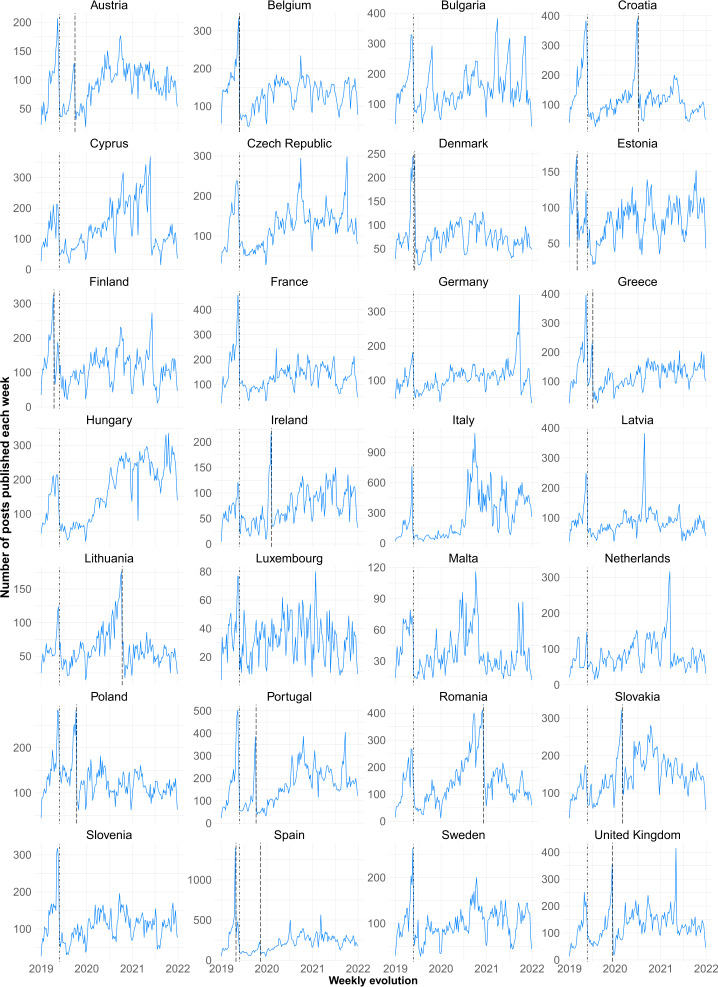
Posts published by country with elections. Each vertical line represents an election

**Figure 16:**
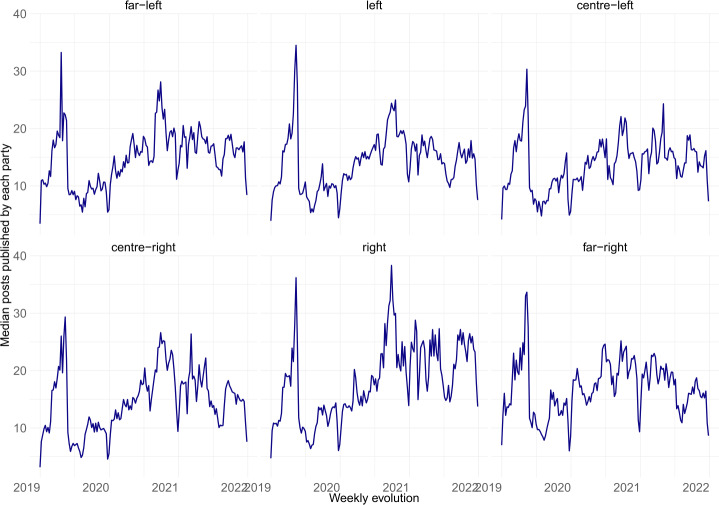
Median number of posts published by different ideology groups

**Figure 17:**
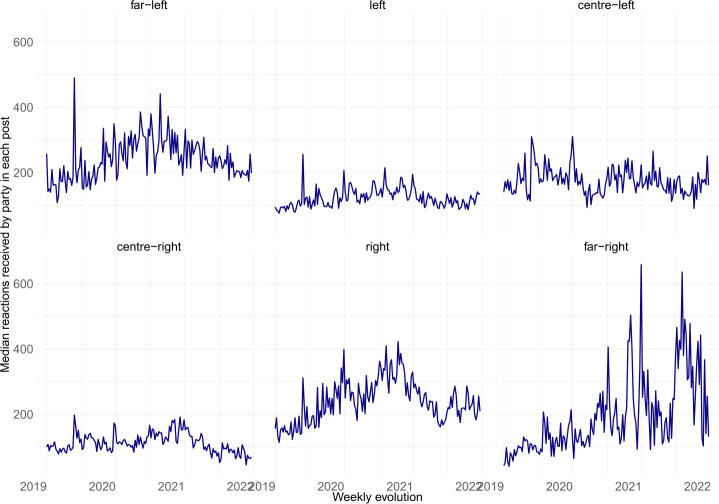
Median number of reactions received in each post by different ideology groups

**Figure 18:**
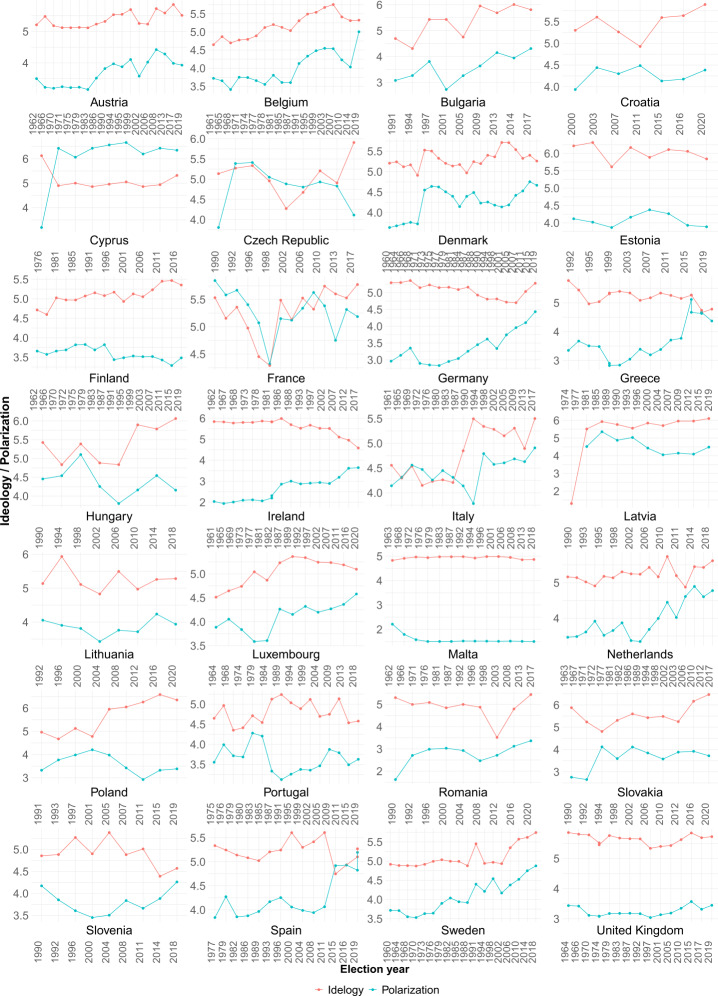
Ideology and Polarization evolution across the EU countries and the United Kingdom

**Figure 19:**
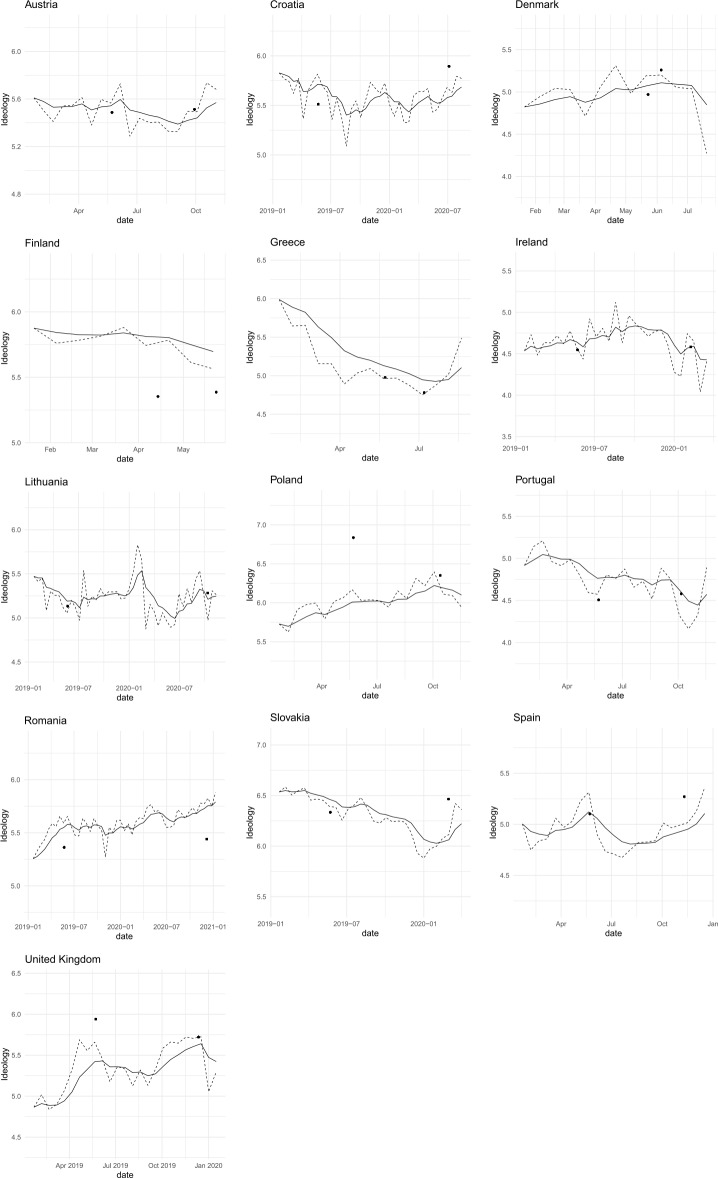
Ideology evolution in European countries

**Figure 20:**
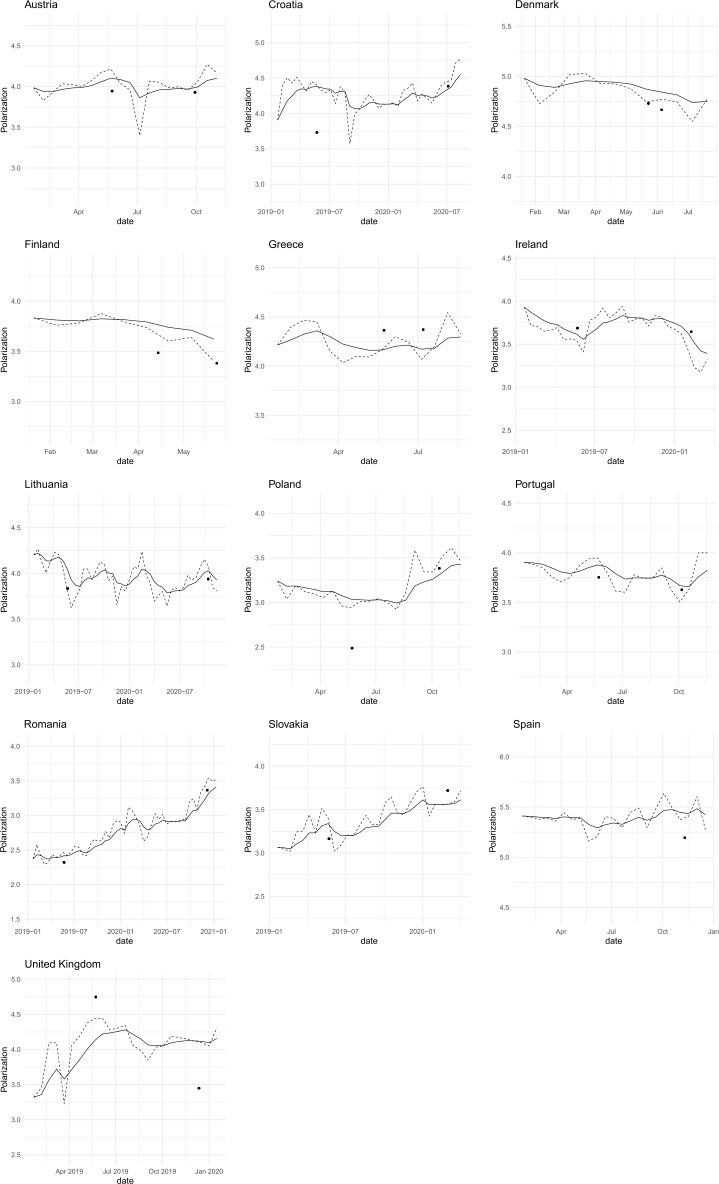
Polarization evolution in European countries

## Data Availability

The processed dataset, ideology and polarization results can be viewed and found at https://eupoliticalbarometer.uc3m.es. Estimated values of ideology and polarization, and the code used to create the models are available to download at https://eupoliticalbarometer.uc3m.es/downloads. The original Facebook dataset is not publicly available in our site. The information can be found at https://facebook.com at each political party official page.

## Appendix

### A.1 Parties publication activity over time

We include in this appendix Fig. 15. This figure displays the number of posts published by the parties of our dataset in the 28 countries considered, to complement the analysis of Sect. 2. The dotted vertical lines represent the European elections that are common for all the countries. In some of the countries (the 15 countries of Sect. 4.1), there is a dashed vertical line displayed. This last line reveals the date of individual parliament elections in that country. When looking at individual countries, we observe a spike in the publication activity before the European elections. For the countries that held individual elections in the period of our study, we see that the same trend happens. There is always a spike before the elections, although sometimes small such as in the case of Spain’s second elections. In all the cases, the post-activity drastically diminishes after electoral periods. Moreover, another interesting result is the cases of the Czech Republic and Italy. We see spikes in Italy at the end of September, and the Czech Republic at the beginning of October, although in these countries no national elections were held. This is due to regional elections in those countries. Therefore, this manifests the ability of our dataset to capture the Facebook activity and intensity of parties in different periods across time.

### A.2 Posts and reactions per ideology

It is interesting to understand the differences in posting activity between the ideologies in the political spectrum. Figure 16 shows the median number of posts published when classifying the parties ideology using the following intervals for their left-right ParlGov score: far-left $0 < 1.5$, left $1.5 < 3.5$ centre-left $3.5 < 5$, centre-right $5 < 6.5$, right $6.5 < 8.5$, far-right $8.5 < 10$. We observe that the median number of posts is relatively consistent independent of the ideology and that the intensity varies mainly due to time events. Moreover, Fig. 17 explores the median number of reactions that each party received per ideology. We see that right ideologies gathered more reactions from Facebook users than left ideologies in the period considered in the dataset.

### A.3 Historical ideology and polarization evolution in the EU and the United Kingdom

In Fig. 18 the evolution of both ideology and polarization is shown for every year that the country held parliamentary elections. We retrieve every instance of election in the ParlGov database of the 27 EU countries and the United Kingdom, then we compute both ideology and polarization for every election. With this image is possible to observe shifts in ideology or polarization, e.g., countries such as Germany, the Netherlands, Spain, or Sweden had an important polarization increase in the last 20 years.

### A.4 Prediction of political parties’ vote share

In this appendix, we evaluate whether the models we have created for estimating political ideology and polarization may provide accurate results in estimating political parties’ vote share. To this end, we have used the weights of our ideology and polarization model to estimate the vote share of each party per country and compute the Mean Absolute Error (MAE), which roughly measures the error in percentage points.

Table 7 shows the MAE when considering the weights of the polarization model and the ideology model in the first and second column respectively. The mean MAE across the evaluated countries is higher than 8 in both cases. Even in some cases like Greece the MAE grows up to 16 points.

**Table 7:** Vote share prediction

Country	MAE $P_{1}$	MAE $I_{1}$
Austria	10.210	10.532
Belgium	3.471	3.072
Croatia	8.887	9.459
Denmark	5.466	5.870
Estonia	11.809	10.069
Finland	5.870	6.453
Greece	11.395	16.562
Ireland	11.807	11.078
Lithuania	3.735	7.451
Poland	8.520	6.382
Portugal	8.857	13.721
Romania	10.604	6.904
Slovakia	2.370	5.570
Spain	9.324	8.231
United Kingdom	9.821	9.634
Mean MAE	8.143	8.732

In summary, these results confirm that the proposed methodology does not lead to accurate enough results to present our models as a good estimator of parties’ vote share.

### A.5 Ideology and polarization evolution in European countries

In Figs. 19 and 20 the evolution for both ideology and polarization for a selection of countries are shown (those with national elections after the European elections). The dashed line represents the values computed using the models $I_{1}$ and $P_{1}$, whereas the solid line represents the EWMA value. The dots indicate the national elections, the first one the EU elections, and the second one the parliamentary election in each country.

### A.6 Lasso regression models

Instead of using the methodology introduced in Sect. 3, (*i.e.*, linear regression models), it would be possible to apply a similar approach but using regularization techniques such as Lasso Regression [45], which could perform feature selection. Therefore using lasso regression we obtained models for both ideology and polarization, which are shown in Table 8. The models were also evaluated in different time windows, and using Cross Validation. Note that the ideology model uses seven features, while the polarization uses five, instead of the two features that use the other models.

**Table 8:** Lasso Regression coefficients

Feature	Ideology coefficients	Polarization coefficients
(Intercept)	1.08272698	2.153661971
posts	.	0.328168828
posts_per_day	0.07648722	0.040113227
reactions	0.11396280	.
reactions_median	0.28914113	0.088896705
reactions_mean	.	.
reactions_median_day	.	.
reactions_median_week	.	.
shares	0.01339832	.
shares_median	.	.
shares_mean	.	.
shares_median_day	.	.
shares_median_week	.	.
comments	.	.
comments_median	−0.17764914	.
comments_mean	.	0.005268904
comments_median_day	.	.
comments_median_week	0.24684151	.
video_views	.	.
video_views_median	.	.
video_views_mean	.	.
video_views_median_day	.	.
video_views_median_week	.	.
followers	.	.
page_likes	0.12555404	0.142732740

The obtained models could be used to try to predict the ideology and polarization in elections of the 2019-2020 period, the results are shown in Table 9. For country ideology, this approach seems to get a slightly better result than the model $I_{1}$ (an RMSE difference of 0.02 points), but it is still worse than the baseline model. The polarization model performs worse than the $P_{1}$ model (RMSE increases 0.068 points). Therefore, the usage of this technique does not improve the results overall.

**Table 9:** Lasso Regression results for parliamentary elections in 2019-2020

	Country Ideology			Dalton Polarization		
Country	Real	Prediction	Error	Real	Prediction	RMSE
Austria	5.512	5.435	0.077	3.928	4.139	−0.211
Belgium	5.324	5.641	−0.317	5.005	4.677	0.328
Croatia	5.894	5.755	0.139	4.385	4.494	−0.109
Denmark	5.260	5.051	0.209	4.666	4.707	−0.041
Estonia	5.842	5.267	0.575	3.886	2.985	0.901
Finland	5.353	5.592	−0.239	3.485	3.594	−0.109
Greece	4.781	4.813	−0.032	4.371	4.246	0.125
Ireland	4.583	4.644	−0.061	3.645	3.367	0.278
Lithuania	5.283	5.059	0.224	3.938	3.273	0.665
Poland	6.351	6.154	0.197	3.383	3.368	0.015
Portugal	4.580	4.407	0.173	3.627	3.616	0.011
Romania	5.441	5.808	−0.367	3.364	3.250	0.114
Slovakia	6.465	6.111	0.354	3.720	3.629	0.091
Spain	5.271	4.960	0.311	5.195	5.326	−0.131
United Kingdom	5.722	5.623	0.099	3.447	3.838	−0.391
	Country Ideology			Dalton Polarization		
RMSE	0.264			0.338		

## Footnotes

1 Note that some of the entries included in the ParlGov dataset correspond to the same party after a party name change or to parties that do not exist anymore.

2 As stated in Sect. 2.1.3 we collected different types of reactions, but we decided to only include the aggregated variables in this section, as the desegregated ones did not improve the predictive performance.

3 Note that we do not include the 2 days prior to the elections since any engagement shown in posts 48h before the elections could be caused by the election result itself. As shown in Fig. 2 the engagement metrics of posts rarely show any relevant increase after 48 hours. Therefore, the 48h after the publication posting has a negligible influence on the popularity and activity metrics, and including them could lead to misleading in the modeling process.

4 We remind we are not using Germany to generate the prediction model because we are missing the FB page from one of the main political parties, i.e, the *SPD*.

## Abbreviations

EU: European Union
UK: United Kingdom
FB: Facebook
PPD: Posts Per Day
SD: Standard Deviation
VIF: Variance inflation factor
RMSE: Root-Mean-Square Error
R^2^: Coefficient of determination
EWMA: Exponentially Weighted Moving Average
